# A novel β-TrCP1/NRF2 interaction inhibitor for effective anti-inflammatory therapy

**DOI:** 10.1186/s12929-025-01157-3

**Published:** 2025-07-11

**Authors:** Ángel J. García-Yagüe, Lucía Cañizares-Moscato, José Antonio Encinar, Eduardo Cazalla, Raquel Fernández-Ginés, Maribel Escoll, Ana I. Rojo, Antonio Cuadrado

**Affiliations:** 1 Department of Biochemistry, School of Medicine, Autonomous University of Madrid (UAM), Madrid, Spain; 2 Instituto de Investigaciones Biomédicas “Sols-Morreale” (CSIC-UAM), C/ Arturo Duperier, 4, 28029 Madrid, Spain; 3 Instituto de Investigación Sanitaria La Paz (IdiPaz), Madrid, Spain; 4 Centro de Investigación Biomédica en Red de Enfermedades Neurodegenerativas (CIBERNED), Madrid, Spain; 5 Experimental Therapeutics Program, Spanish National Cancer Research Centre (CNIO), Madrid, Spain; 6 Institute of Research, Development, and Innovation in Biotechnology of Elche (IDiBE) and Molecular and Cell Biology Institute (IBMC), Miguel Hernández University (UMH), 03202 Elche, Alicante, Spain

**Keywords:** NRF2, β-TrCP1, Protein–protein interaction-inhibitor, Inflammation, Liver

## Abstract

**Background:**

Non-communicable chronic diseases are characterized by low-grade inflammation and oxidative stress. Extensive research has identified the transcription factor NRF2 as a potential therapeutic target. Current NRF2 activators, designed to inhibit its repressor KEAP1, often exhibit undesirable side effects. As an alternative approach, we previously developed PHAR, a protein–protein interaction inhibitor of β-TrCP1/NRF2, which promotes NRF2 activation. Using the same in silico screening platform, we have now identified a novel compound, P10. This small molecule selectively interferes with the β-TrCP1/NRF2 interaction, leading to NRF2 stabilization and transcriptional activation of its target genes in a β-TrCP1-dependent manner, demonstrating promising effects in a liver model of acute inflammation.

**Methods:**

After an in silico screening of ∼1 million compounds, including molecular docking analysis, ADMET evaluation, and molecular dynamics simulations, we identified and characterized a novel small molecule, P10, which inhibits β-TrCP1/NRF2 interaction. The compound was validated using luciferase reporter assays, co-immunoprecipitation, and ubiquitination experiments. The specificity of P10 was assessed by comparing NRF2 signatures in wild-type and *Nrf2*-null cells. The impact of NRF2 activation induced by P10 was investigated by evaluating its antioxidant and anti-inflammatory responses against tert-butyl hydroperoxide and lipopolysaccharide, respectively. Finally, wild-type and *Nrf2*-null mice were administered P10 intraperitoneally at a dose of 20 mg/kg daily for five consecutive days. Four hours before sacrifice, all animals received a lipopolysaccharide (LPS) injection at 10 mg/kg.

**Results:**

P10 selectively disrupts the interaction between β-TrCP1 and NRF2, thereby inhibiting β-TrCP1-mediated ubiquitination of NRF2 and leading to the upregulation of NRF2 target genes. Additionally, P10 mitigates oxidative stress induced by tert-butyl hydroperoxide and reduces pro-inflammatory markers in an NRF2-dependent manner in macrophages treated with lipopolysaccharide. In a preclinical model of liver inflammation, P10 specifically targets the liver, significantly attenuating lipopolysaccharide-induced inflammation through the activation of NRF2. This is demonstrated by decreased expression of inflammatory cytokine genes and a reduction in F4/80-stained liver macrophages. Notably, this anti-inflammatory effect is absent in *Nrf2*-knockout mice, confirming its NRF2-dependent mechanism of action.

**Conclusions:**

P10 emerges as a promising NRF2 activator by selectively disrupting the β-TrCP1/NRF2 interaction, highlighting its potential as a therapeutic agent for diseases presenting acute liver inflammation.

**Graphical Abstract:**

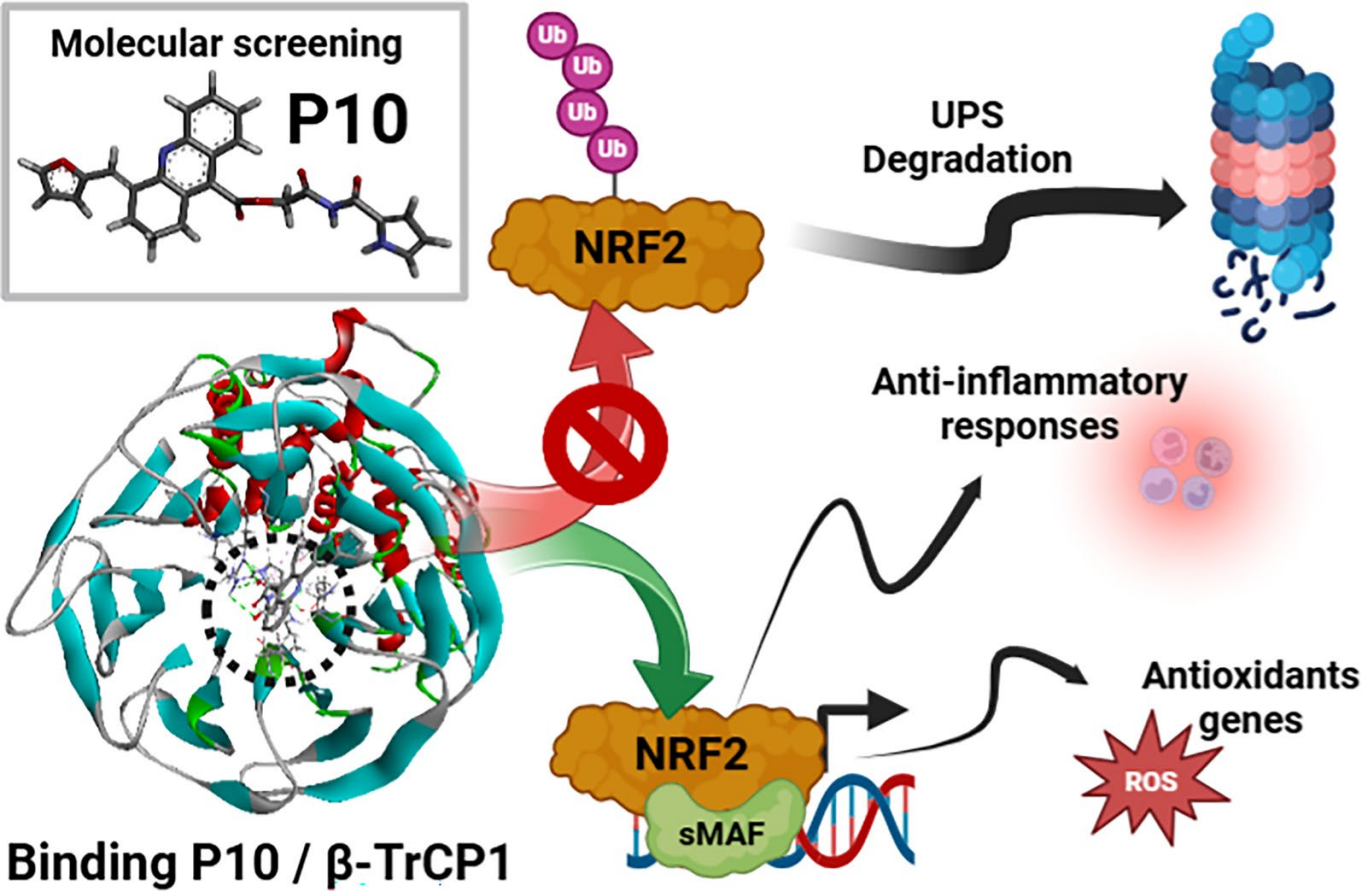

**Supplementary Information:**

The online version contains supplementary material available at 10.1186/s12929-025-01157-3.

## Introduction

NRF2 (Nuclear factor erythroid 2-related factor 2) is a cap‘n’collar (CNC) basic-region leucine zipper (bZIP) transcription factor that plays a crucial role in cellular homeostasis [1, 2]. It regulates the basal and inducible expression of over 250 genes containing the Antioxidant Response Element (ARE, 5′-TGACNNNGC-3′) in their regulatory regions [2, 3]. These genes encode diverse cytoprotective proteins involved in detoxification, biotransformation, antioxidant defense, inflammation, and intermediary metabolism, collectively forming a protective transcriptional program [4]. While NRF2 is widely recognized for its role in regulating oxidative stress and promoting cytoprotective responses, it also exerts immunomodulatory functions. NRF2 induces the expression of anti-inflammatory genes such as CD36, MARCO, and IL-17D [5–7], while repressing pro-inflammatory genes like IL-6 and IL-1β [8]. Additionally, NRF2 contributes to immune regulation by controlling reactive oxygen species (ROS) levels, which influence NF-κB signaling [9–11], and by inhibiting immune cell infiltration through the regulation of VCAM and MMP9 expression (12–14).

NRF2 protein stability is regulated at least by two E3 ubiquitin ligase adaptors: KEAP1 and β-TrCP. The latter mediates NRF2 degradation following prior phosphorylation by glycogen synthase kinase-3β (GSK-3β). Under homeostatic conditions, KEAP1 forms a homodimer that binds NRF2 at two amino acid motifs with differing affinities: a low-affinity DLG motif and a high-affinity ETGE motif. This interaction presents NRF2 for ubiquitination by the CUL3/RBX1 (Cullin-3/RING-box protein 1) complex, leading to proteasomal degradation [15, 16]. As a result, NRF2 remains at low basal levels under basal physiological conditions [17, 18]. KEAP1 contains several redox-sensitive cysteine residues that can be modified by oxidants or electrophilic compounds [19, 20]. Under oxidative stress, these modifications impair KEAP1-mediated NRF2 degradation, allowing newly synthesized NRF2 to accumulate, translocate into the nucleus, heterodimerize with small MAF proteins, and activate its target genes. The pharmacological inhibition of KEAP1 has been a major area of research, leading to the development of omaveloxolone and dimethyl fumarate (DMF)—approved KEAP1 inhibitors used to treat Friedreich's Ataxia, psoriasis, and multiple sclerosis, respectively [21–24]. However, many electrophilic NRF2 activators lack specificity for KEAP1, as they modify cysteines on various proteins, which can potentially cause unintended cellular effects. Protein–Protein Interaction inhibitors (PPI-inh) have emerged to overcome this limitation, as a new class of NRF2 activators for targeting the NRF2/KEAP1 interaction [25–27]. These compounds aim to selectively prevent NRF2 binding to KEAP1, offering a more targeted approach to NRF2 activation.

Our group identified a phosphorylation-dependent degradation motif (phosphodegron) within the Neh6 domain of NRF2, which serves as a target for GSK-3β-mediated phosphorylation [28, 29]. Once phosphorylated, this motif is recognized by the E3 ligase adaptor β-TrCP (β-transducin repeat-containing protein). Together, GSK-3β and β-TrCP facilitate NRF2 degradation via the CUL1/RBX1 complex [28–31]. Given its pivotal role in NRF2 regulation, the β-TrCP/NRF2 interaction represents a promising therapeutic target. We previously identified PHAR, the first protein–protein interaction inhibitor (PPI-inh) reported to disrupt the β-TrCP/NRF2 complex, leading to NRF2 activation [32, 33]. Although β-TrCP-mediated NRF2 degradation remains an underexplored pathway for pharmacological NRF2 activation, our findings with PHAR highlight its potential as a viable molecular target. Conversely, PHAR presents certain limitations regarding its ADMET (Absorption, Distribution, Metabolism, Excretion, and Toxicity) properties. Specifically, PHAR fails in one of Lipinski’s Rule of Five criteria, exceeding the molecular weight threshold of 500 Da. This higher molecular weight contributes to permeability issues and crossing cell membranes, negatively impacting the human body's pharmacokinetic properties and overall drug-like behavior. To overcome these limitations, we expanded our search for novel compounds that comply with these criteria while effectively modulating this pathway. Thus, P10 emerged as a strong alternative candidate from these efforts and is now undergoing experimental validation.

In this paper, we present P10, a novel PPI-inh developed by our group, which demonstrates superior inhibition of the β-TrCP1/NRF2 interaction. P10 exhibits strong mechanistic protection against lipopolysaccharide (LPS)-induced inflammation in macrophages and effectively mitigates acute liver inflammation in a mouse model.

## Materials and methods

An additional description of some methods is presented in the Supplemental Material.

### Cell culture and reagents

MCF-7 c32^ARE−Luc^ and mouse embryonic fibroblasts (MEFs) were cultured in Dulbecco's modified Eagle's medium (DMEM) supplemented with 10% fetal bovine serum (FBS, HyClone, CH30160.03) with 80 μg/ml gentamicin (Normon Laboratories). Raw264.7 macrophage cells were cultured in Roswell Park Memorial Institute medium (RPMI1640, Sigma-Aldrich, R6504) with 10% fetal bovine serum (FBS) and 80 μg/ml gentamicin. MCF-7 c32^ARE−luc^ cells were kindly provided by Prof. C. Roland Wolf (University of Dundee, UK). *Keap1*^+/+^ and *Keap1*^−/−^ MEFs were kindly provided by Prof. Ken Itoh (Centre for Advanced Medical Research, Hirosaki University Graduate School of Medicine, Hirosaki, Japan). *Nrf2*^+/+^ and *Nrf2*^−/−^ MEFs were obtained from respective wildtype and *Nrf2*-knockout C57BL/6 mice kindly provided by Prof. Masayuki Yamamoto (Department of Medical Biochemistry, Tohoku University Graduate School of Medicine, Sendai, Japan). Other reagents, including 3-(4,5-dimethylthiazol-2-yl)-2,5-diphenyltetrazolium (MTT), lipopolysaccharide from Escherichia coli O111:B4 (LPS, L4391), MG132 (C2211), SB216763 (S3442), and tBHP (tert-Butyl hydroperoxide, 458,139), were purchased from Sigma-Aldrich. Regarding reagents for signaling assays, we include R, S-sulforaphane (SFN, LKT Laboratories, Inc. ID S8044), tert-butylhydroquinone (tBHQ, Fluka 19,986), and LY294002 (TOCRIS, 934,389–88-5). LY294002, SB216763, tBHQ, and P10 were dissolved in dimethyl sulfoxide (DMSO). The final concentration of DMSO in cell culture was less than 0.2%.

### Animals and treatments

All experimental procedures were performed according to the *Guide for the Care and Use of Laboratory Animals* and had been previously approved by the Autonomous Community of Madrid (PROEX 105/18). All efforts were made to minimize animal suffering and to reduce the number of animals used. Animals were housed under controlled conditions (22 ± 1 °C, 55–65% humidity, 12 h light–dark cycle) with free access to water and standard laboratory chow. For HPLC analysis of organ exposure to P10, 6-month-old C57BL/6 mice were used for each experimental condition (n = 5). Mice were randomly divided into two groups that received an intraperitoneal (i.p.) injection of either vehicle (DMSO: Tween-80: Saline 0.9%; 20:15:65) or P10 (20 mg/kg body weight dissolved in the vehicle). This dose was chosen based on the highest concentration that could be effectively used within the solubility range permitted by our selected vehicle formulation. This dose allowed for proper compound delivery without precipitation or formulation issues, and appreciable toxicity during the experiment. After 2 h, livers were collected for biochemical analyses and HPLC. For chronic treatments, mice were randomly divided into two groups that received an i.p. injection of either vehicle or P10 (20 mg/kg) daily. After 5 days, the liver, brain, and lung were obtained for biochemical analyses. For induction of acute liver inflammation, 6-month-old *Nrf2*^+/+^ and *Nrf2*^−/−^ mice were randomly divided into two groups (n = 5) per genotype. They received an i.p. injection of either vehicle (groups 1 and 3) or P10 (groups 2 and 4) as indicated in the chronic regimen for five days. Then, mice were treated with an i.p. injection of either saline (groups 1 and 2) or 10 mg/kg body weight LPS (groups 3 and 4). Mice were sacrificed after 4 h, and livers were collected for biochemical analyses.

### Computational approaches

This section is nearly identical to that described previously in a publication from our group [32]. In this instance, the 1P22 crystallographic structure for β-TrCP1-SKP1-β-CATENIN was used. ADMET predictions, molecular docking, and molecular dynamics simulations were performed in DataWarrior v5.0.0 software and YASARA structure software, or detailed in the aforementioned paper [32]. The only notable difference is that the current version of YASARA (v24.4.10) allows for calculating Gibbs free energy variation using Vina 1.2.5.

### Plasmids

The vector HA-Ubiquitin was provided by Dr. Tadashi Nakagawa (Division of Cell Proliferation, ART, Tohoku University Graduate School of Medicine, Sendai, Japan). A plasmid encoding pcDNA3-Flag-β-TrCP1 was kindly provided by Dr. Tomoki Chiba (Department of Molecular Biology, University of Tsukuba, Japan). The expression vectors, pcDNA3.1/V5His-mNRF2^ΔETGE^, pcDNA3.1/V5His-mNRF2^ΔETGE^-6S6A, pcDNA3.1/V5His-mNRF2^ΔETGE^-4S4A and pcDNA3.1/V5His-mNRF2^ΔETGE^-2S2A, have been previously described [34].

### Luciferase assays

MCF-7 c32^ARE−LUC^ cells were seeded on 24-well plates (75,000 cells per well) and incubated with P10 and SFN for 16 h. Then, cells were lysed and assayed with a luciferase assay system (Promega) according to the manufacturer's instructions [35]. Relative light units were measured in a GloMax 96 microplate luminometer with dual injectors (Promega). Each sample was measured from at least triplicate samples.

### Cell viability assessment by MTT reduction

In live cells but not in dead ones, the tetrazolium ring of 3-(4,5-dimethylthiazol-2-yl)-2,5-diphenyltetrazolium bromide (MTT) can be reduced by active dehydrogenases to produce a formazan precipitate [32]. At the end of the experiments, cells were washed three times with phosphate-buffered saline (PBS) followed by the addition of MTT (0.125 mg/ml) and incubation for 1 h at 37 °C. Thereafter, the media was removed and DMSO was added to each well to dissolve the formazan precipitate for 30 min, thereby determining the relative number of alive cells. An aliquot (100 μl) of the supernatants was analyzed in 96-well multiwell plates at 550 nm in a VERSAmax microplate reader (Molecular Devices).

### High-performance liquid chromatography (HPLC)

The mouse liver samples (∼80 mg) were treated with 0.5 ml methanol, grinded in a potter, and centrifuged. The supernatant was collected and filtered through a 0.45 μm PTFE microfilter (Fisherbrand). The samples were first analyzed by HPLC–UV and the eluted peaks were further analyzed by HPLC–MS afterwards. As an internal standard, P10 was prepared in methanol and analyzed by HPLC–MS.

### Lentiviral vector production and infection

The pseudotyped lentivirus particles were produced in HEK293T cells employing shβ-TrCP1 (NM_009771 TRCN-0000012807) and shβ-TrCP2 (NM_134015 TRCN-0000231303) (Sigma-Aldrich; MISSION shRNA), and scrambled RNA vector (Addgene; shCtrl 1864). Briefly, a mixture of 6 μg of envelope plasmid (pMD2G 12259, Addgene), 6 μg of packaging plasmid (psPAX2 12,260, Addgene) and 10 μg of transfer vector was prepared in OPTIMEM media (Thermo Fisher Scientific) and transfected into HEK293T cells (2 × 106 cells/100 mm dishes), using Lipofectamine and Plus Reagent (Thermo Fisher Scientific) according to the manufacturer's protocol. Lentivirus-containing supernatant was harvested and passed through a 0.45 μm filter. Cells were infected in the presence of 4 μg/ml polybrene (Sigma-Aldrich) and selected with 1 μg/ml puromycin (Sigma-Aldrich) for 5 days.

### Immunoblotting

This protocol was performed as described in [36]. Briefly, cells were washed once with cold PBS and lysed with lysis buffer (TRIS pH 7.6 50 mM, 400 mM NaCl, 1 mM EDTA, 1 mM EGTA, and 1% SDS). The samples were sonicated and precleared by centrifugation, and cell lysates were resolved in SDS-PAGE and transferred to Immobilon-P membranes (Millipore, Billerica, MA). These membranes were analyzed using the primary antibodies indicated in Supplementary Table 1, and the appropriate peroxidase-conjugated secondary antibodies. Proteins were detected by enhanced chemiluminescence (GE Healthcare).

### Ubiquitination assay

MCF-7 c32^ARE−Luc^ cells were seeded on 60-mm dishes (5 × 10^5^ cells per dish), cultured for 16 h, and co-transfected with expression plasmids for HA-Ubiquitin (HA-Ub), pcDNA3-Flag-β-TrCP1 and either pcDNA3.1/V5His-mNRF2^ΔETGE^, pcDNA3.1/V5His-mNRF2^ΔETGE^-6S6A, pcDNA3.1/V5His-mNRF2^ΔETGE^-4S4A or pcDNA3.1/V5His-mNRF2^ΔETGE^-2S2A, using Lipofectamine 2000 (Invitrogen) according to manufacturer recommendations (see details in Supplemental Material).

### Co-immunoprecipitation assay

Previously described in [36]. MCF-7 c32^ARE−Luc^ cells were seeded on 60-mm dishes (5 × 10^5^ cells per dish) and cultured for 16 h. Subsequently, the cells were co-transfected with expression plasmids for pcDNA3-Flag-β-TrCP1 together with either pcDNA3.1/V5His-mNRF2^ΔETGE^ or pcDNA3.1/V5His-mNRF2^ΔETGE^-6S6A, using Lipofectamine 2000 (Invitrogen) (see details in Supplemental Material).

### Analysis of mRNA levels by real-time quantitative PCR

Total RNA was extracted using TRIzol reagent according to the manufacturer's instructions (Invitrogen). Reverse transcription and quantitative PCR were done as detailed elsewhere [37]. Primer sequences are shown in Supplementary Table 2. Data analysis was based on the ΔΔCT method with normalization of the raw data to housekeeping mouse/human genes *Vcl/VCL* and *Gapdh/GAPDH* (Applied Biosystems). All PCRs were performed in triplicate.

### Determination of reactive oxygen species by flow cytometry

MCF-7 c32^ARE−Luc^ cells were incubated for 1 h at 37 °C with 2 μM dihydroethidine (DHE; ThermoFisher Scientific) and then detached from the plate, washed once with cold PBS, and analyzed immediately. Intracellular reactive oxygen species (ROS) were detected in a FACSCanto II (BD Biosciences) flow cytometer according to DHE oxidation, which emits orange fluorescence (BP 575/24 nm).

### Histological analysis

Livers were post-fixed with 4% paraformaldehyde for 24 h and then submerged in 70% ethanol. Next, they were embedded in paraffin, cut into 5 μm-thick sections, and stained with hematoxylin and eosin (H&E). For immunohistochemical analyses, sections were deparaffinized and rehydrated in water, and antigen retrieval was carried out by incubation with citrate buffer pH 6.0 at 50 °C for 30 min. Endogenous peroxidase and nonspecific antibody reactivity were blocked by treatment with 3% H_2_O_2_ at room temperature for 10 min. The sections were then incubated for 16 h at 4 °C with the corresponding peroxidase-conjugated primary antibody (diluted in PBS containing 1% normal goat serum) F4/80 (1:150, Serotec) and developed with 3,3′-diaminobenzidine (DAB). The sections were counterstained with hematoxylin, dehydrated in ethanol, then in xylol, and then mounted in DePex (Thermo Fisher Scientific). Negative controls with goat normal serum, replacing the primary antibody, were used. Densitometric quantification was done using macros of the ImageJ software [32].

### Statistical analysis

Unless otherwise indicated, all experiments were performed at least 3 times and all data presented in the graphs are the mean of at least 3 independent experiments. Data are presented as mean ± S.D. (standard deviation). Statistical differences between groups were assessed using GraphPad Prism 8 software by the unpaired Student’s t-test. One and two-way analyses of variance with post- Bonferroni’s test were used for multiple comparisons. A p-value ≤ 0.05 was considered statistically significant. Statistically significant differences are indicated in the figures (***p values < 0.001, ** < 0.01 and * < 0.05) (^###^p values < 0.001, ^##^ < 0.01 and ^#^ < 0.05).

## Results

### In silico identification of a small molecule putatively targeting the β-TrCP1/NRF2 interaction

**Fig. 1:**
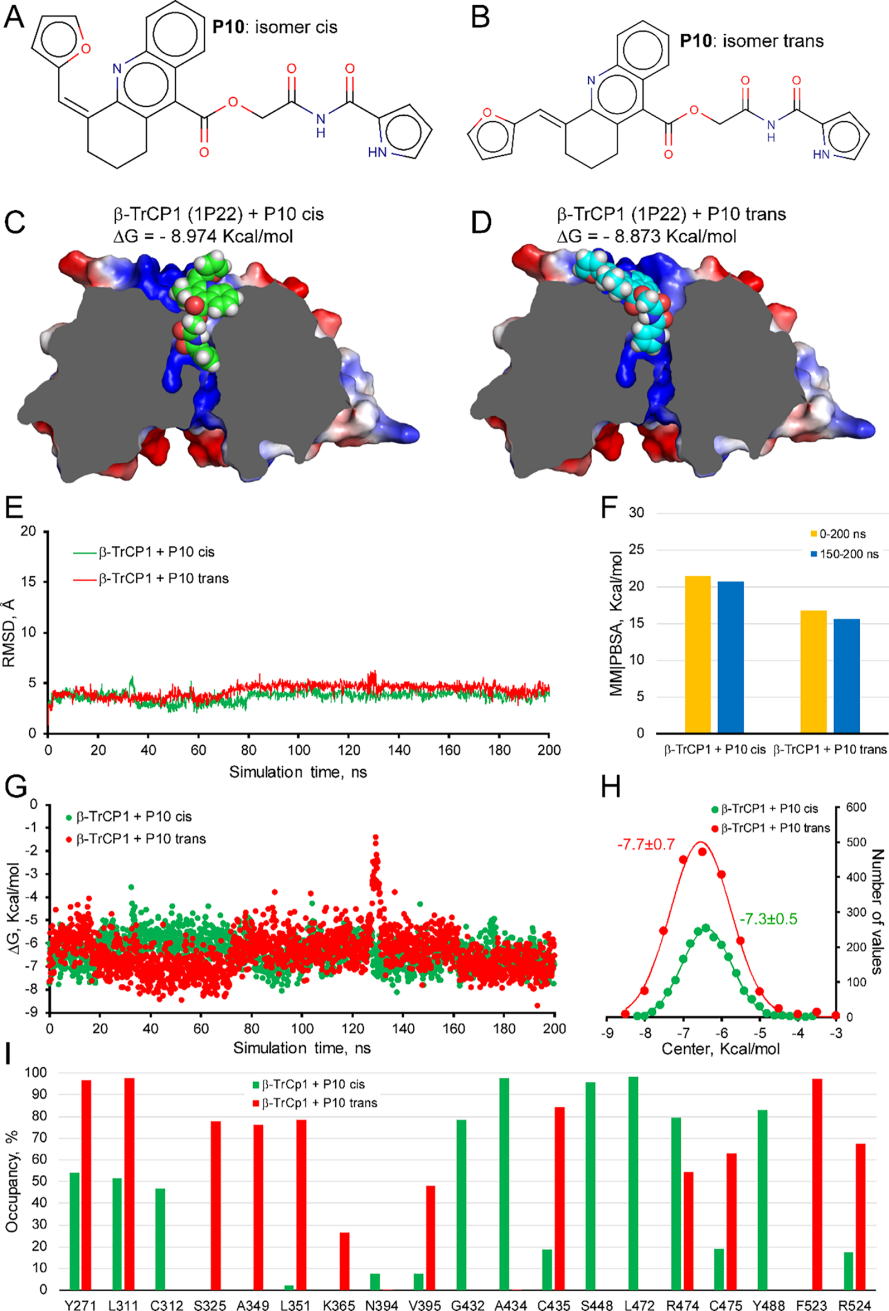
Selection of P10 as a candidate disruptor of the β-TrCP1-NRF2 interaction, based on molecular docking and dynamics simulations. **A**, **B** Structures of the cis and trans isomers of P10, respectively. **C**, **D** Low energy of P10 cis- and trans molecules docked to the surface of β-TrCP1, in the WD40 domain (PDB code 1P22). The ΔG values (calculated with YASARA software using Vina v1.2.5) are provided, where more negative values indicate stronger binding of P10 to the β-TrCP1 protein. **E** Molecular dynamics simulation of trajectories of the P10 isomers bound to β-TrCP1 during a 200 ns. **F** Calculated MM|PBSA free energy values for the P10-β-TrCP1 isomer complex. MM|PBSA calculations from YASARA yield positive values when strong and stable binding is predicted, whereas negative values suggest weak or no binding. **G** ΔG values were calculated for each snapshot generated during the 200 ns of MD simulation, with more negative values reflecting stronger binding of P10 to the protein. The frequency plot of the ΔG values for each simulation exhibits a Gaussian distribution. **H** ΔG values (mean ± S.D., calculated from the Gaussian fit) for each P10-protein complex. **I** Occupancy time of β-TrCP1 amino acids interacting with each P10 isomer over the 200 ns MD simulation

Using the selection criteria previously described in [32], this study identified another weak NRF2 activator and docked it to the WD40 propeller of β-TrCP1. This compound exists in two isomeric forms: 2-oxo-2-(1H-pyrrole-2-carboxamido)ethyl (Z)-4-(furan-2-ylmethylene)-1,2,3 and 2-oxo-2-(1H-pyrrole-2-carboxamido) ethyl (E)-4-(furan-2-ylmethylene)-1,2,3,4-tetrahydroacridine-9-carboxylate. For simplicity, in this study, we will call P10 the racemic mix (Fig. 1A and B). The core structure of P10 is 2, 4-tetrahydroacridine-9-carboxylate. Fig. 1C and D show the binding of P10 docked to the WD40 domain of β-TrCP1, close to the channel of WD40 and partially overlapped with the NRF2 binding reported in [29]. Images of interaction in the sagittal plane among P10 and WD40 of β-TrCP1 suggest that P10 seems to be drain-plug shaped (Suppl. Fig. S1 A–D). The Gibbs free energy variation values are − 8.98 kcal/mol for the cis-isomer and − 8.87 kcal/mol for the trans-isomer. Molecular dynamics simulations were performed for 200 ns on the P10-β-TrCP1 complexes generated by molecular docking (Fig. 1C and D). Figure 1E shows the trajectory of both isomers bound to the protein, with no shifts beyond 5 Å from the initial pose determined by the docking simulation, indicating stable binding of each isomer to the protein. Figure 1F shows the calculated solvation binding energy values (MM|PBSA) for the protein–ligand interaction, which remains stable over the estimated time (the last 50 ns of the 200 ns simulation). These values are comparable to, or slightly higher than, those previously calculated for PHAR [32]. Additionally, we calculated the Gibbs free energy variation for each of the 2000 snapshots taken during the molecular dynamics simulation (Fig. 1G). This parameter follows a Gaussian distribution (Fig. 1H), allowing us to determine average ΔG values binding of − 7.3 ± 0.5 kcal/mol for the P10-cis and − 7.7 ± 0.7 kcal/mol for the P10-trans. The orientation of both isomers in their interaction with β-TrCP1 is similar, with the extreme pyrrole ring entering the protein channel in both cases. Curiously, there are only minor differences in the ΔG values of the two isomers. One possible explanation may be the position of the front furane group linker to aromatic rings of acridine of P10 seems to have an interaction with any amino acid of β-propeller β-TrCP1 in the trans, but not in cis isomers (Suppl. Fig. S1A–D). However, analysis of the “fingerprint” generated on the amino acids of the β-TrCP1 binding site reveals clear differences (Fig. 1I). The most notable differences are observed in the hydrogen bonding patterns of each isomer with β-TrCP1. In the P10-cis isomer, hydrogen bonds are formed with the residues Tyr271, Leu311, and Arg524. In contrast, the P10-trans isomer interacts with Arg474, Phe523, and Arg524, resulting in clearly different occupancy rates (Fig. 1I). Furthermore, the majority of interactions during the simulation are hydrophobic for both isomers, and the patterns of the amino acids involved differ, with Ser325, Ala349, Leu351, Val395, Cys435, Cys475, and Phe523 for P10-trans and Cys312, Gly432, Ala434, Ser448, Leu472, and Tyr488 for P10-cis. Thus, both isomers might be interfering in the interaction of NRF2 with β-TrCP1 in this region, thereby preventing the proteasomal degradation of NRF2. However, it is worth noting that while interaction with key residues of β-TrCP1 is predicted for both isomers, mainly Arg^474^, Arg^524^, Leu^351^, Asn^394^, and Ala^434^ [29], the score associated with these interactions may vary, implying that they may not be equally effective in inhibition of the interaction with these residues (Suppl. Fig. S1E and F).

### P10 increases NRF2 protein levels and its target genes.

**Fig. 2:**
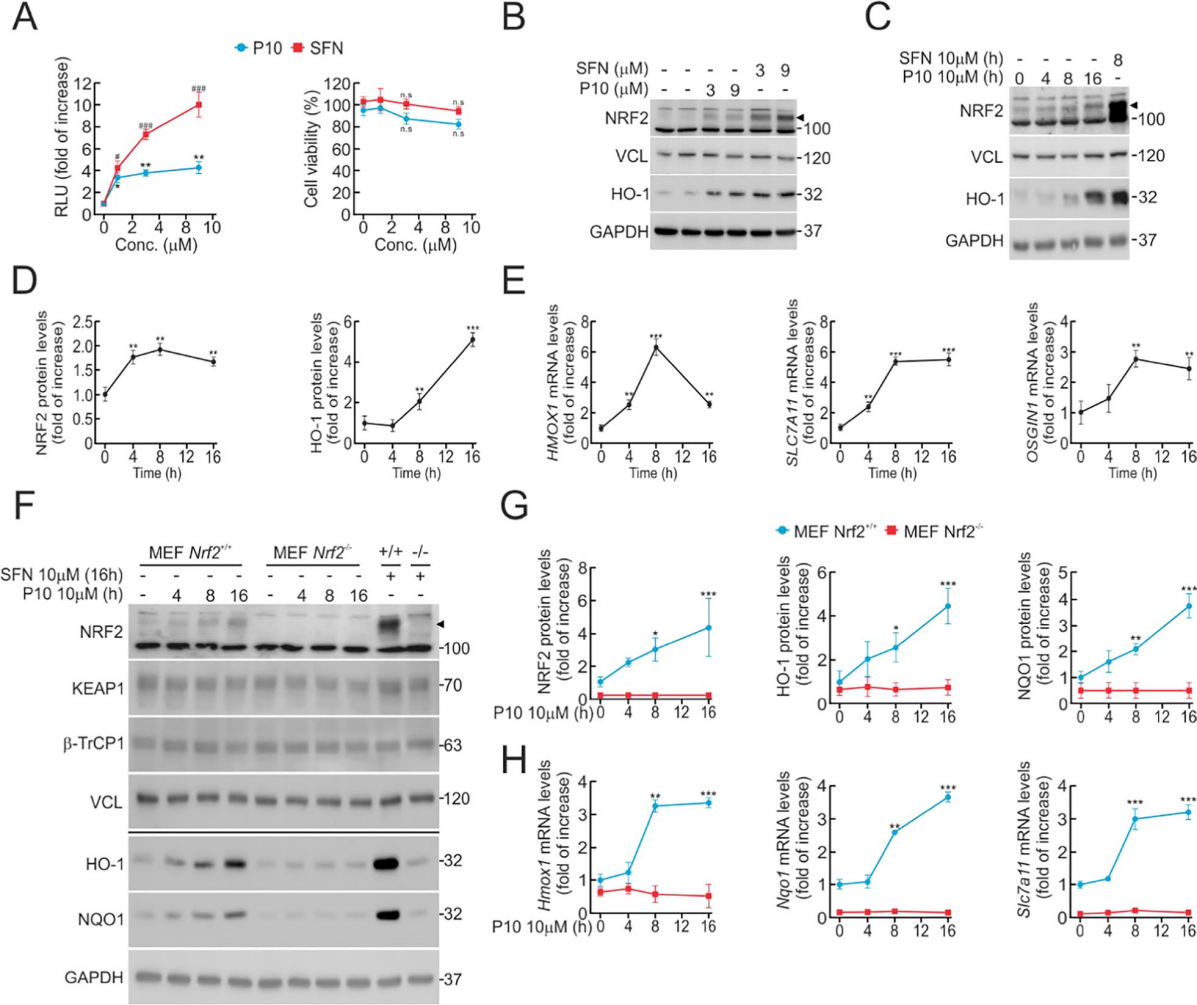
Evaluation of P10 as an NRF2 inducer. **A** MCF-7 c32^ARE−LUC^ reporter cells were maintained under low-serum conditions (16 h, 1% FBS) and then subjected to the indicated P10 concentrations or 10 μM SFN, as a positive control. 0.1% DMSO was used as a vehicle. Luciferase activity was measured after 16 h of treatment. Data are mean ± S.D. (n = 4). *p < 0,05; **p < 0.01; #p < 0,05; ###p < 0.001 vs. vehicle according to a one-way ANOVA test. MTT assay, performed to determine the cell viability of P10 and SFN in low-serum starved (1% FBS) cells. Data are mean ± S.D. (n = 4). **B** MCF-7 c32^ARE−LUC^ cells under low-serum conditions (16 h, 1% FBS) were subjected to the indicated P10 concentrations and SFN, as a positive control, for 16 h. Representative immunoblots of NRF2 (arrowhead) and HO-1, VCL, and GAPDH as a loading control. The NRF2 blot shows a strong unspecific band that is shown as an additional loading control. **C** Representative immunoblots of NRF2 (arrowhead) and HO-1, VCL, and GAPDH as a loading control from low-serum conditions (16 h, 1% FBS) MCF-7 c32^ARE−LUC^ were submitted to 10 μM P10 for the indicated times, and SFN as a positive control. **D** Densitometric quantification of NRF2 and HO-1 protein levels from representative immunoblots of C expressed as a ratio of VCL and GAPDH, respectively. Data are mean ± S.D. (n = 3). **p < 0.01; ***p < 0.001 vs. vehicle according to a one-way ANOVA test. **E** Low-serum conditions (16 h, 1% FBS) MCF-7 c32^ARE−LUC^ were subjected to 10 μM P10 for the indicated times. Transcript levels of *HMOX1*, *SLC7A11*, and *OSGIN1* were determined by qRT-PCR and normalized by the geometric mean of GAPDH and VCL levels. Data are mean ± S.D. (n = 3). **p < 0.01 ***p < 0.001 according to a one-way ANOVA test. **F** Representative immunoblots of NRF2 (arrowhead), HO-1, NQO1, KEAP1, β-TrCP1, VCL, and GAPDH as a loading control from low-serum conditions (16 h, 1% FBS) MEFs from *Nrf2*-wildtype (*Nrf2*^+/+^) and *Nrf2*-knockout (*Nrf2*^−/−^) mice subjected to 10 μM P10 for the indicated times. **G** Densitometric quantification of NRF2, HO-1, and NQO1 protein levels from representative immunoblots from F, expressed as a ratio of VCL and GAPDH, respectively. Data are mean ± S.D. (n = 3). *p < 0,5; **p < 0,01; ***p < 0.001 vs vehicle of *Nrf2*^+/+^ according to a two-way ANOVA test. **H** mRNA levels of several ARE-genes were determined after 10 μM P10 for the indicated times, by qRT-PCR and normalized by the geometric mean of *Gapdh* and *Vcl* levels. Data are mean ± S.D. (n = 3). **p < 0,01; ***p < 0.001 vs *Nrf2*^+/+^ according to a two-way ANOVA test

The ability of P10 to activate NRF2 was first examined using the MCF-7 c32ARE-LUC cell line, which contains 8 tandem repeats of the ARE sequence driving the expression of the firefly luciferase gene [35]. To achieve a significant activation of the GSK-3/β-TrCP1 axis, cells were maintained under low-serum conditions (16 h, 1% FBS) [28], followed by P10 treatment (16 h, 3 and 9 μM) and then analyzed for luciferase activity. As shown in Fig. 2A, P10 produced a ∼fourfold increase of the luciferase ARE reporter, which was about half the effect observed with the canonical KEAP1 inhibitor SFN at 9 µM [38]. The analysis of cell viability with MTT did not show significant cellular toxicity at any of the concentrations used (Fig. 2A). We also evaluated the effect of P10 on activation of the endogenous NRF2 and one of its main target genes HO-1, and the levels of both proteins were increased (Fig. 2B). Additionally, in time-course experiments, P10 increased NRF2 protein levels after 4 h treatment and maintained plateau values at least for 16 h (Fig. 2C and D). The *bona-fide* transcript targets of NRF2 (ARE-genes), *HMOX1*, *SLC7A11*, and *OSGIN1* were analyzed in parallel. As shown in Fig. 2E, P10 increased the transcript levels of the three ARE-genes from 4 to 16 h.

To corroborate that P10 induces the expression of ARE-genes in an NRF2-dependent manner, MEFs from *Nrf2*-wildtype (*Nrf2*^+/+^) or *Nrf2*-knockout (*Nrf2*^−/−^) mice were incubated with 10 μM P10 at different time points (4, 8, and 16 h). In *Nrf2*^+/+^ MEFs, P10 produced a significant increase in HO-1 and NQO1proteins that was not observed in *Nrf2*^−/−^ MEFs (Fig. 2F and G). Moreover, in *Nrf2*^−/−^ MEFs, P10 did not significantly induce the expression of three robust NRF2 targets, *Hmox1*, *Nqo1*, and *Slc7a11* (Fig. 2H). These results indicate that P10 activates NRF2, which is responsible for the increase in the expression of its target genes, therefore excluding other mechanisms.

### P10 activates NRF2 in KEAP1-deficient cells.

**Fig. 3:**
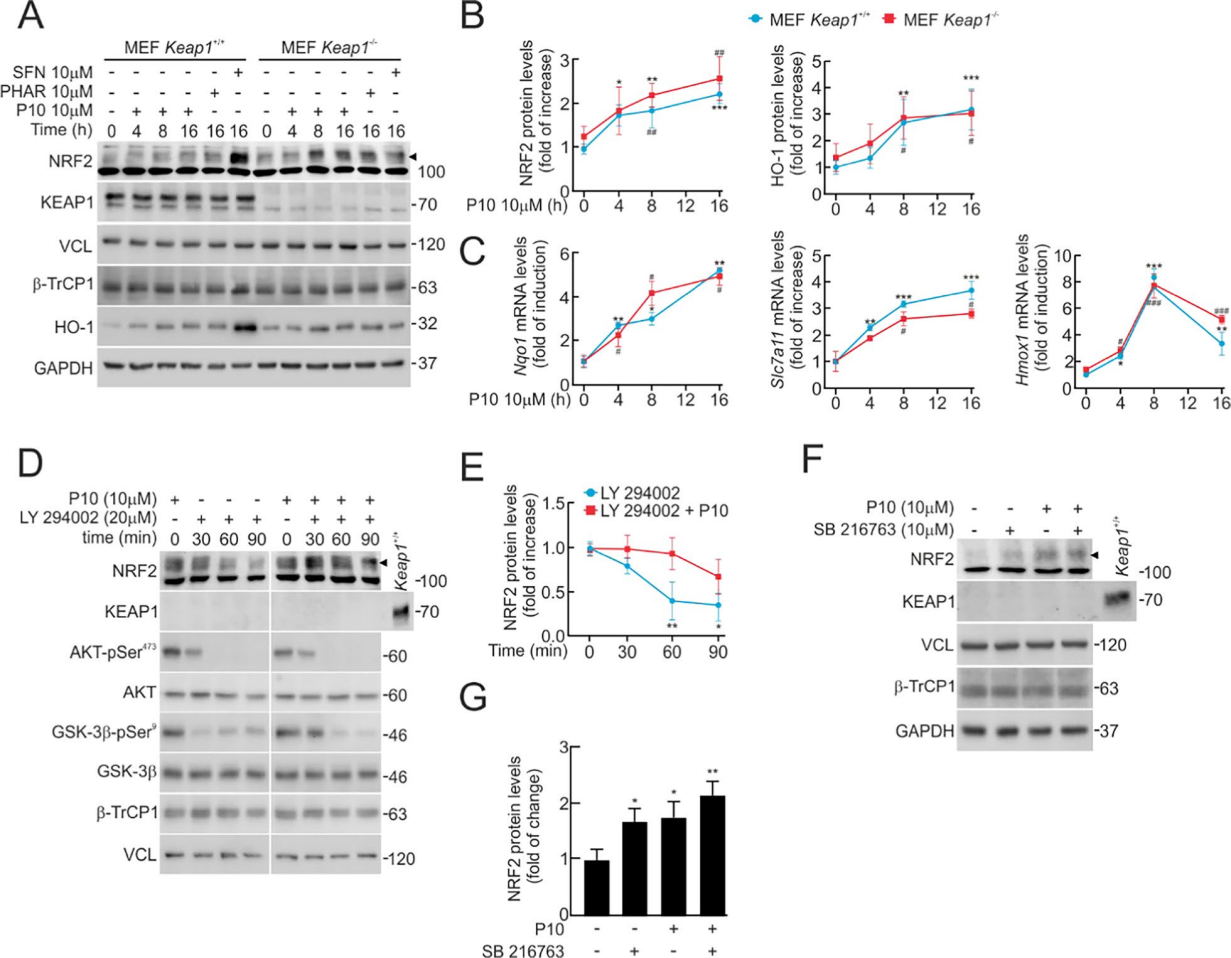
P10 induces NRF2 signature in a KEAP1-independent but PI3K/AKT/GSK3β-dependent manner. **A** Representative immunoblots of NRF2 (arrowhead), HO-1, β-TrCP1, KEAP1, VCL, and GAPDH as a loading control from low-serum conditions (16 h, 1% FBS) MEFs from *Keap1*-wildtype (*Keap1*^+/+^) and *Keap1*-knockout (*Keap1*^−/−^) mice subjected to 10 μM P10 for the indicated times, and 10 μM SFN and 10 μM PHAR, as a positive control. **B** Densitometric quantification of NRF2 and HO-1 protein levels from representative immunoblots from **A**, expressed as a ratio of VCL and GAPDH, respectively. Data are mean ± S.D. (n = 3). *p < 0.05; **p < 0.01; ***p < 0.001; #p < 0,05 vs point 0 according to a two-way ANOVA test. **C** mRNA levels of ARE-genes were determined after 10 μM P10 for the indicated times, by qRT-PCR and normalized by the geometric mean of *Gapdh* and *Vcl* levels. Data are mean ± S.D. (n = 3). *p < 0.05; **p < 0.01; ***p < 0.001; #p < 0,05; ###p < 0.001 vs point 0 according to a two-way ANOVA test. **D** Representative immunoblots of NRF2, AKT-pSer^473^, AKT, GSK-3β-pSer^9^, GSK-3β, KEAP1, β-TrCP1, and VCL as a loading control. Low-serum conditions (16 h, 1% FBS) *Keap1*^−/−^ MEFs were subjected to the vehicle (0.1% DMSO) or 10 μM of P10 for 16 h. Then, cells were treated with 20 μM LY294002 for the indicated times. **E** Densitometric quantification of NRF2 protein levels from representative immunoblots from **D**, expressed as a ratio of VCL. Data are mean ± S.D. (n = 3). *p < 0.05 vs LY2940002 at point 0, according to a two-way ANOVA test. **F** Representative immunoblots of NRF2 (arrowhead), KEAP1, β-TrCP1, VCL, and GAPDH as a loading control. *Keap1*^−/−^ MEFs were low-serum conditions (16 h, 1% FBS) and then subjected to 10 µM P10, 10 µM SB216763 (GSK-3 inhibitory agent), or to both treatments for 8 h. **G** Densitometric quantification of NRF2 protein levels from representative immunoblots from F, expressed as a ratio of GAPDH and VCL. Data are mean ± S.D. (n = 3) *p < 0.05; **p < 0.01; ***p < 0.001 according to Student t-test

Since NRF2 is mainly regulated by the E3-ubiquitin ligase adapter KEAP1, we deemed it necessary to determine if P10 might act via inhibition of KEAP1. We addressed this question by comparing the effects of P10 on NRF2-targets in MEFs from *Keap1*-wildtype (*Keap1*^+/+^) and *Keap1*-knockout (*Keap1*^−/−^) mice (Fig. 3A and B). As expected, we found higher levels of NRF2 and HO-1 proteins at the resting state in *Keap1*^−/−^ MEF cells compared to *Keap1*^+/+^ MEF cells, as previously reported in [32, 39]. While 10 μM SFN, used as a positive control for KEAP1 inhibition, led to NRF2 activation only in *Keap1*^+/+^ MEFs, a time-course incubation with 10 μM P10 revealed a similar NRF2 and HO-1 protein increase and of *Hmox1*, *Nqo1* and *Scl7a11* transcript levels in both cell lines. The effect of P10 was similar to that of a recently described β-TrCP/NRF2 inhibitor, PHAR (10 μM, 16 h) (Fig. 3C).

### P10 does not alter signaling cascades.

The PI3K/AKT/GSK-3β signaling cascade has been reported to play a role in β-TrCP1-mediated regulation of NRF2 [29, 40, 41]. To investigate if P10 might alter this pathway, we first used the PI3K inhibitor LY294002. The inhibition of PI3K results in GSK-3β activation, leading to the phosphorylation of NRF2 at its Neh6 domain and promoting its degradation via interaction with β-TrCP [40]. To eliminate potential alternative mechanisms mediated by KEAP1, we used *Keap1*^−/−^ MEFs. Cells were pre-treated with 10 μM P10 or vehicle for 16 h and then treated with 20 μM LY294002 for the indicated times. After 60 min, LY294002 caused a notable decrease in AKT-pSer^473^ (inhibition) and GSK-3β-pSer^9^ (activation), which correlated with a reduction in NRF2 protein levels (Fig. 3D). Conversely, P10 partially rescued NRF2 from the effect to LY294002 (Fig. 3D and E). To further analyze the putative participation of GSK-3β in the P10 response, we used the GSK-3β inhibitor SB216763 [28]. P10 increased NRF2 levels to the same extent as SB216763. Moreover, when cells were subjected to both SB216763 and P10 drugs, NRF2 levels were increased to a similar extent as with any of them alone (Fig. 3F and G), suggesting that both drugs target the same pathway.

Some electrophiles like tert-butyl hydroquinone (tBHQ) may also activate NRF2 through modification of signaling events. Thus, inhibition of the lipid and protein phosphatase PTEN, leading to GSK-3β activation through inhibition of the PI3K/AKT axis, results in NRF2 phosphorylation, creating a β-TrCP1 recognition site for ubiquitin–proteasome degradation [28, 29]. We compared the effect of P10 and tBHQ as controls in the MCF-7 c32^ARE−LUC^ cell line. As expected, 10 µM of both P10 and tBHQ increased NRF2 levels over time with different dynamics (Suppl Fig. S2A and D). tBHQ increased pSer^473^-AKT- and pSer^9^-GSK-3β levels, indicative of their activation and inhibition, respectively, but this effect was not observed with P10, suggesting that P10 does not alter this pathway (Suppl Fig. S2B and D).

MAPKs modify NRF2 activity by poorly defined mechanisms [42–44]. To determine if P10 could impact these pathways, we treated the MCF-7 c32^ARE−LUC^ cell line with P10 and tBHQ as a control. We found that tBHQ increased the phosphorylation of the three MAPKs, phospho-p38 (pThr^180^/pTyr^182^), phospho-ERK1/2 (pThr^202^/pTyr^204^), and phospho-SAPK/JNK (pThr^183^/pTyr^185^) (Suppl Fig. S2C and S2D). However, P10 did not modify them, suggesting that P10 induces NRF2 without activating these kinases (Suppl Fig. S2C and S2D).

### P10 increases NRF2 protein levels in a β-TrCP1-dependent manner.

**Fig. 4:**
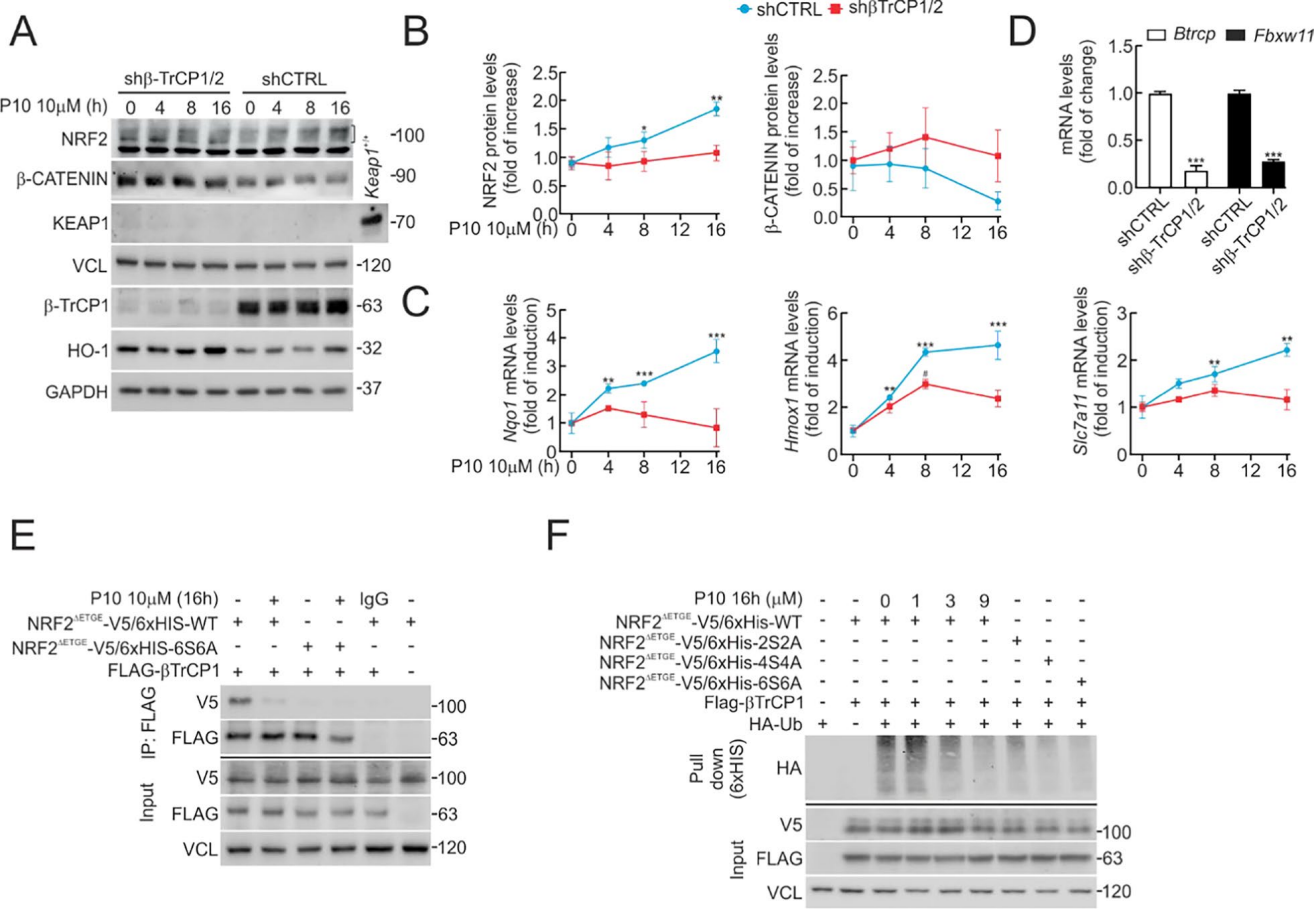
P10 increases NRF2 protein levels in a β-TrCP1-dependent-manner. **A** Representative immunoblots from control (shCTRL) and β-TrCP knocked-down (shβ-TrCP1/2) MEFs that were submitted to 10 μM P10 for the indicated times, including the following proteins: NRF2 (bracket), HO-1, β-CATENIN, KEAP1, β-TrCP1, and VCL and GAPDH as a loading control. **B** Densitometric quantification of NRF2 and β-CATENIN protein levels from representative immunoblots from A, normalized with GAPDH and VCL. Data are mean ± S.D. (n = 3). *p < 0,05; **p < 0.01 vs. shCTRL at point 0 according to a one-way ANOVA test. **C** Expression of three NRF2-regulated genes in shCTRL vs. shβ-TrCP1/2 *Keap1*^−/−^ MEFs. Cells were submitted to 10 μM P10 for the indicated times, and transcript levels were determined by qRT-PCR and normalized by the geometric mean of *Gapdh* and *Vcl* levels. Data are mean ± S.D. (n = 3). **p < 0.01; ***p < 0.001 vs. shCTRL at point 0 according to a two-way ANOVA test. **D** Knockdown of *Btrc* (encoding β-TrCP1) and *Fbxw11* (encoding β-TrCP2). *Keap1*^−/−^ MEFs were transduced with control lentivirus encoding shCTRL or a combination of two lentiviruses encoding sh-*Btrc* and sh-*Fbxw11*. After 5 days, transcript levels of *Btrc* and *Fbxw11* were determined by qRT-PCR and normalized by the geometric mean of *Gapdh* and *Vcl*. Data are mean ± S.D. (n = 3). ***p < 0.001 vs. shCTRL according to a Student's t-test. **E** MCF-7 c32^ARE−LUC^ cells were cotransfected with the indicated plasmids or with an empty vector. After transfection (5 h), cells were in low-serum conditions (16 h, 1% FBS) and subjected to 10 µM P10. One-fifth of the whole-protein lysate was used to control for protein expression, and it was blotted with V5, FLAG, and VCL. The rest of the protein lysates were immunoprecipitated with anti-FLAG antibodies and immunoblotted with anti-FLAG and anti-V5. **F** MCF-7 c32^ARE−LUC^ cells were transfected with the indicated plasmids. After transfection (5 h), cells were in low-serum conditions (16 h, 1% FBS) and subjected to P10 for the indicated concentrations. An affinity-purified His-tagged fraction (His-Pull-down) was blotted with an anti-HA antibody, and a whole-cell lysate (input) was blotted with V5, FLAG, and VCL as loading control. As controls of ubiquitin bound to NRF2, we carried out one with just co-transfected NRF2^ΔETGE^-V5/6xHis with Flag-β-TrCP1 without HA-Ub for validating the recognition of specific HA antibody smear-band, and an additional control employed just HA-Ub transfected for evaluating unspecific HA-Ub binding to Probond beads

Our *in-silico* analysis is consistent with P10 being a β-TrCP1/NRF2 interaction inhibitor. To get insight into this possibility, we employed a knock-down approach in MEFs from *Keap1*^−/−^ mice. Since two mammalian β-TrCP paralogues exist, β-TrCP1 and β-TrCP2 [30], we knocked-down both isoforms. Cells were transduced with lentiviral vectors encoding sh-β-TrCP1 and sh-β-TrCP2 for 5 days (Fig. 4A–D). This protocol yielded a decrease of > 90% for β-TrCP1 (*Btrc*) and > 80% for β-TrCP2 (*Fbxw11*) mRNA levels (Fig. 4D) compared to shCTRL-infected cells. Accordingly, protein levels of the canonical substrate, β-CATENIN, were increased in sh-*Btrc* and sh-*Fbxw11* infected cells compared to control.

In shCTRL-infected cells, P10 led to an increase in NRF2 protein levels as early as 4 h and sustained for at least 16 h (Fig. 4A and B). However, P10 did not affect β-CATENIN levels, suggesting that P10 is not capable of displacing β-CATENIN from its interaction with β-TrCP1 (see Discussion and [45]). In contrast, in β-TrCP1/2 knocked-down cells, NRF2 levels were higher and were not further increased by P10 (Fig. 4A and B). Additionally, the response to P10 was also impaired in the expression of three NRF2-target genes, *Hmox1*, *Nqo1*, and *Slc7a11* (Fig. 4C).

To gain further information about the contribution of P10 in disrupting the association between NRF2 and β-TrCP1, we conducted a co-immunoprecipitation assay. MCF-7 c32^ARE−LUC^ cells were co-transfected with NRF2^ΔETGE^-V5/6xHis, which lacks the high-affinity ETGE domain of NRF2 for KEAP1 binding, or NRF2^ΔETGE^-V5/6xHis-6S6A, which, in addition, lacks the motif for recognition by β-TrCP1 as a control. Cells were maintained under low-serum conditions (16 h, 1% FBS), co-transfected with Flag-β-TrCP1 and pre-treated with P10 (10 µM, 16 h). Subsequently, the lysed cells were immunoprecipitated using an anti-FLAG antibody. NRF2 with an intact Neh6 domain (NRF2^ΔETGE^-V5/6xHis) was pulled down with β-TrCP1 (Fig. 4E). In contrast, P10 abolished this interaction (Fig. 4E). As expected, P10 did not affect the NRF2^ΔETGE^-V5/6xHis-6S6A mutant, which does not interact with β-TrCP1 (Fig. 4E).

Next, we sought to determine if P10 inhibits β-TrCP1-mediated ubiquitination of NRF2, employing the MCF-7 c32^ARE−LUC^ cell line. Cells are maintained under low-serum conditions (16 h, 1% FBS) and co-transfected with Flag-β-TrCP1, HA-Ub, and NRF2^ΔETGE^-V5/6xHis. Then, we pulled down NRF2 with the nickel column and measured the amount of HA-Ub bound to NRF2. A reduction in ubiquitin bound to NRF2 was found in the NRF2 mutants that do not bind β-TrCP1 (Fig. 4F) [28, 29]. However, NRF2^ΔETGE^-V5/6xHis exhibited a significant level of ubiquitination, and following treatment with different concentrations of P10 for 16 h, we observed a dose-dependent decrease (Fig. 4F).

### P10 reduced tBHP-induced oxidative stress.

**Fig. 5:**
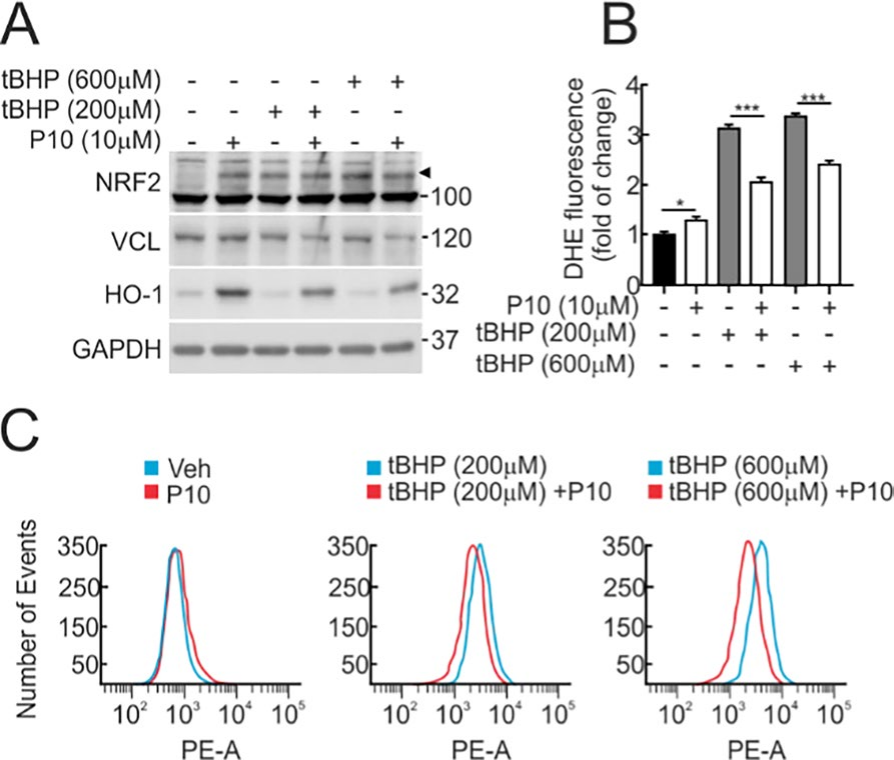
P10 attenuates redox dysregulation. **A** Representative immunoblots of NRF2 (arrowhead), HO-1, VCL, and GAPDH as a loading control from low-serum conditions (16 h, 1% FBS) MCF-7 c32^ARE−LUC^ that were pre-treated with 10 μM P10 for 16 h and then submitted to tert-butyl hydroperoxide (tBHP) as indicated for 3 h. **B**, **C** Flow cytometry analysis of tert-butyl hydroperoxide-induced intracellular ROS production measured by 2 μM hydroethydine (HE) fluorescent probe (BP 575/24 nm). A representative sample of 10,000 cells is shown for each condition. Data are mean ± S.D. (n = 3). *p < 0.05; ***p < 0.001 vs. P10 according to a Student's t-test

The next goal was to analyze whether P10 might exert protection against a strong oxidant insult, such as tert-Butyl Hydroperoxide (tBHP). Low-serum grown (16 h, 1% FBS) MCF-7 c32^ARE−LUC^ cells were incubated with P10 (10 μM, 16 h) and then treated with 200 or 600 μM tBHP for 3 h. As anticipated, P10 caused an upregulation of the levels of NRF2 and the target gene product HO-1 (Fig. 5A). The levels of reactive oxygen species (ROS) were analysed by flow cytometry in parallel cell cultures incubated with 2 μM dihydroethidine (DHE) for 60 min. As shown in Fig. 5B and C, pre-incubation with P10 significantly attenuated the DHE signal in response to both tBHP concentrations. These results suggest that P10 mitigates ROS dysregulation.

### P10 attenuates LPS-induced inflammation in macrophages

Considering the well-established role of NRF2 in the resolution of inflammation [46], we tested the potential anti-inflammatory effect of P10 in lipopolysaccharide (LPS)-treated Raw264.7 mouse macrophages (Fig. Suppl 3 of supplemental material). As expected, 50 ng/ml LPS induced an inflammatory response in vehicle-treated cells after 3 h, evidenced by the increase in the protein levels of pre-IL1β and COX-2. By contrast, P10 led to an expected increase of NRF2 and HO-1 protein levels, along with a significant attenuation of these inflammatory markers (Suppl Fig. S3A and S3B). Similarly, the induction of mRNA levels of inflammatory markers *Il1b*, *Ptgs2*, *Il6*, and *Tnf* was also attenuated in P10-treated cells (Suppl Fig. S3C).

**Fig. 6:**
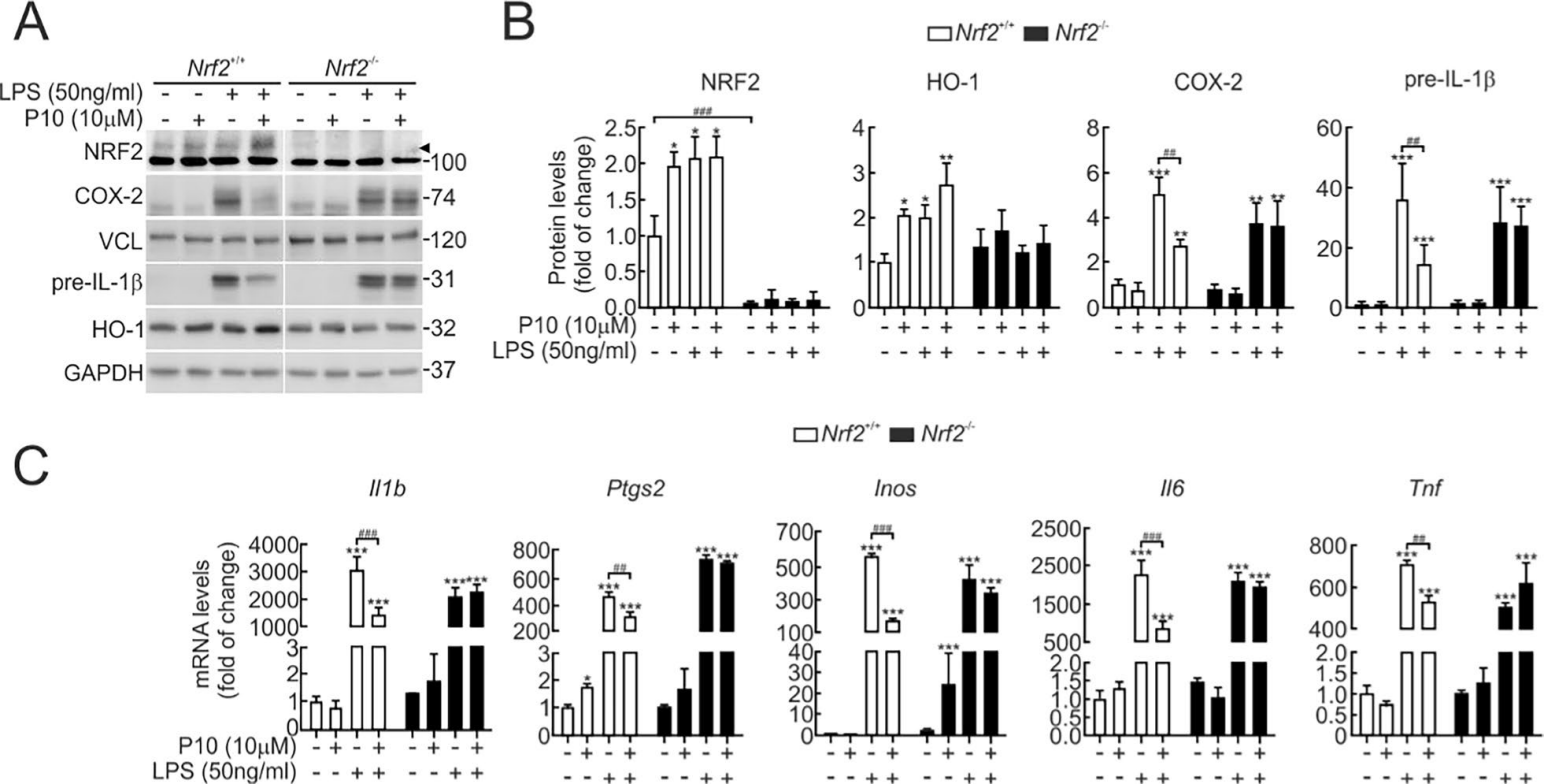
P10 decreases the inflammatory response in an NRF2-dependent manner. Low-serum conditions (16 h, 1% FBS) peritoneal macrophages were pre-treated with 10 µM P10 for 16 h and then incubated with 50 ng/ml LPS for 3 h. **A** Representative immunoblots of NRF2 (arrowhead), HO-1, COX-2, pre-IL-1β, GAPDH, and VCL as a loading control. **B** Densitometric analysis of NRF2, HO-1, COX-2, and pre-IL-1β protein levels from representative immunoblots from A, normalized with GAPDH or VCL. Data are mean ± S.D. (n = 3). *p < 0.05; **p < 0,01; ***p < 0,001 vs. vehicle or LPS treatment, ##p < 0,01; ###p < 0.001 according to the comparison bar, as indicated, according to a one-way ANOVA test. **C** Transcript levels of *Il1b*, *Ptgs2*, *Inos*, *Il6*, and *Tnf* were determined by qRT-PCR and normalized by the average of *Gapdh* and *Vcl*. Data are mean ± S.D. (n = 3). *p < 0.05; **p < 0,01; ***p < 0.001 vs. vehicle or LPS treatment; ##p < 0,01; ###p < 0.001 according to the comparison bar, as indicated according to a one-way ANOVA test

We also determined if P10 required NRF2 to elicit the anti-inflammatory response by using peritoneal macrophages isolated from *Nrf2*^+/+^ and *Nrf2*^−/−^ mice. Pre-treatment of P10 (10 μM, 16 h) was followed by an LPS incubation (50 ng/ml) for 3 h. We found that in *Nrf2*^+/+^ macrophages, P10 attenuates the LPS-induced expression of pre-IL-1β and COX-2 proteins (Fig. 6A and B), and *Il1b*, *Ptgs2*, *Inos*, *Il6*, and *Tnf* transcripts (Fig. 6C), but not in *Nrf2*^−/−^ macrophages. These results show that P10 exerts an anti-inflammatory effect in an NRF2-related manner.

### P10 increases NRF2 protein levels in the liver.

**Fig. 7:**
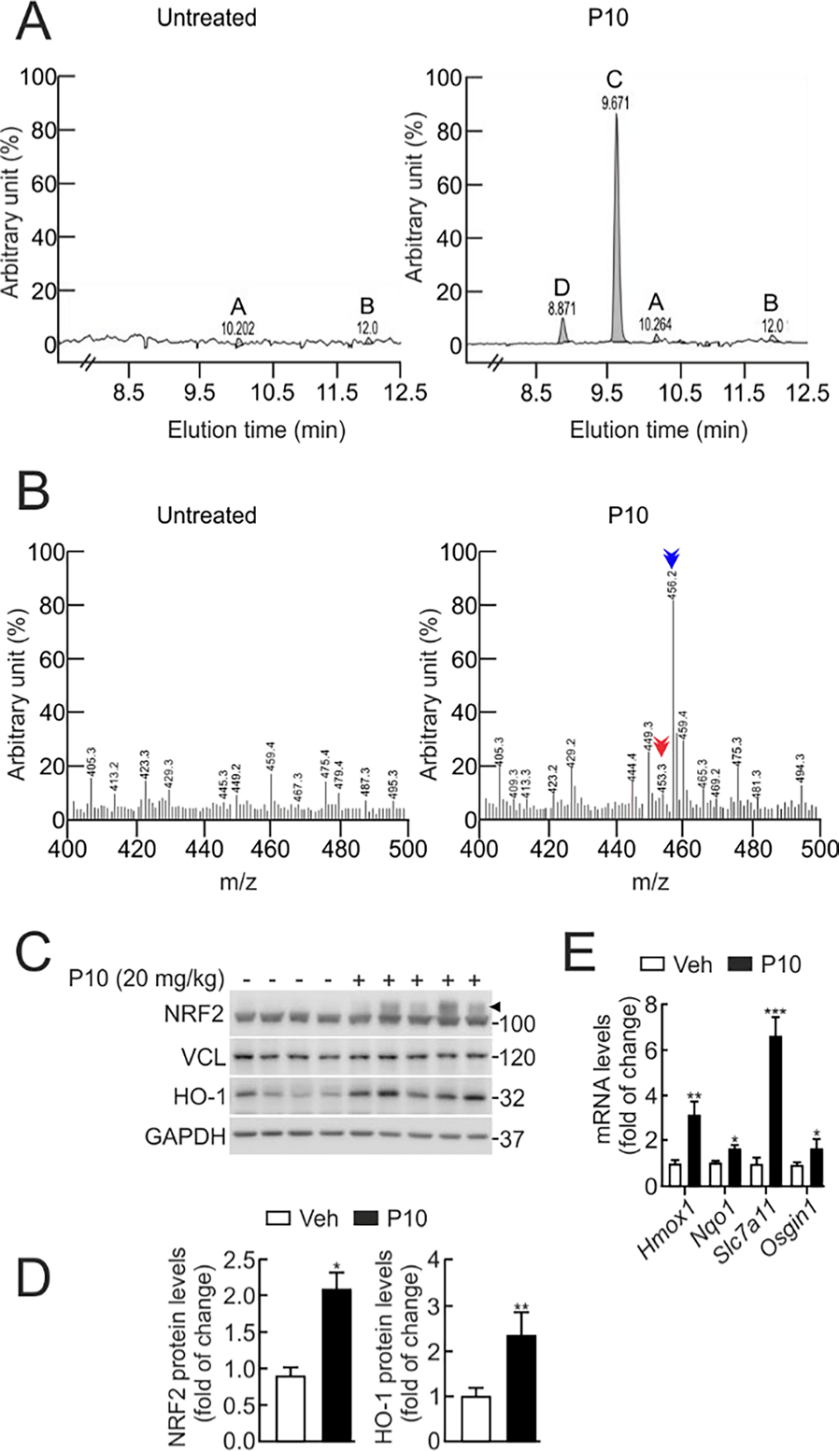
P10 activates NRF2 in the liver. C57Bl/6 male mice received one intraperitoneal (i.p.) injection of 20 mg/kg P10 daily for 5 days, and their livers were analyzed 2 h after the last treatment. **A** Analysis by HPLC–UV of liver extracts comparing vehicle (Tween-80 + PBS, 1:7) vs. P10-treated mice (Tween-80 + PBS, 1:7). **B** Left graph, analysis by HPLC–MS liver vehicle elution. Right graph, analysis by HPLC–MS of peak C detected in the liver of P10-treated mice. Peak D could be a product metabolism of P10 from the liver. Note that the identification of a 456 Da molecule (blue arrow) and a 453 Da (red arrow) in liver P10-treated does not appear in liver-vehicle elution, corresponding to the P10 molecular weight. **C** Representative immunoblots of NRF2 (arrowhead), HO-1 and GAPDH, and VCL as a loading control from liver extracts of each mouse from the vehicle and P10-treated mice i.p. with 20 mg/kg. **D** Densitometric quantification of NRF2 and HO-1 levels from C normalized with GAPDH or VCL. Data are mean ± S.D. (n = 3). *p < 0.05; **p < 0.01 vs. vehicle according to a Student’s t-test. **E** mRNA levels of ARE-genes were determined from liver extracts of the vehicle and P10-treated mice, by qRT-PCR and normalized by the geometric mean of *Gapdh* and *Vcl* levels. Data are mean ± S.D. (n = 3). *p < 0.05; **p < 0.01; ***p < 0.001 vs vehicle according to a Student's t-test

An in vivo model was used to assess whether P10 regulates NRF2 activity in the liver of C57BL/6 mice. Animals received daily intraperitoneal injections of either P10 (20 mg/kg) or vehicle (Tween-80: PBS, 1:7) for five consecutive days. On the fifth day, 2 h after the last administration, we measured P10 levels by HPLC and their biological activity. Comparison of the HPLC–UV profile in the liver of P10 vs. vehicle-treated mice demonstrated two common non-specific peaks at approximately 10.2 and 12.02 min of elution time (Fig. 7A, letters A and B). Interestingly, there were two peaks present specifically in the P10-treated group that eluted at 9.6 and 8.8 min (Fig. 7A, letters C and D). The HPLC–MS analysis of the peak at 9.6 min revealed the existence of a major compound with a molecular weight of 456 Da, consistent with P10 (Fig. 7B, right graph, blue arrow). The peak at 8.8 min, with a molecular weight of 453 Da, may correspond to a metabolic product of P10 (Fig. 7B, right graph, red arrow). In parallel, hepatic NRF2 protein levels exhibited a significant increase upon administration of P10 (20 mg/kg) (Fig. 7C–E). The accumulation of NRF2 matched with higher levels of HO-1 and NQO1, within this time frame. We also observed the induction of the transcriptional NRF2 signature (*Hmox1*, *Nqo1*, *Osgin1,* and *Slc7a11*) (Fig. 7C–E). These results indicate that P10 reaches the liver through i.p. administration and activates NRF2.

### P10 attenuates acute liver inflammation in response to LPS

**Fig. 8:**
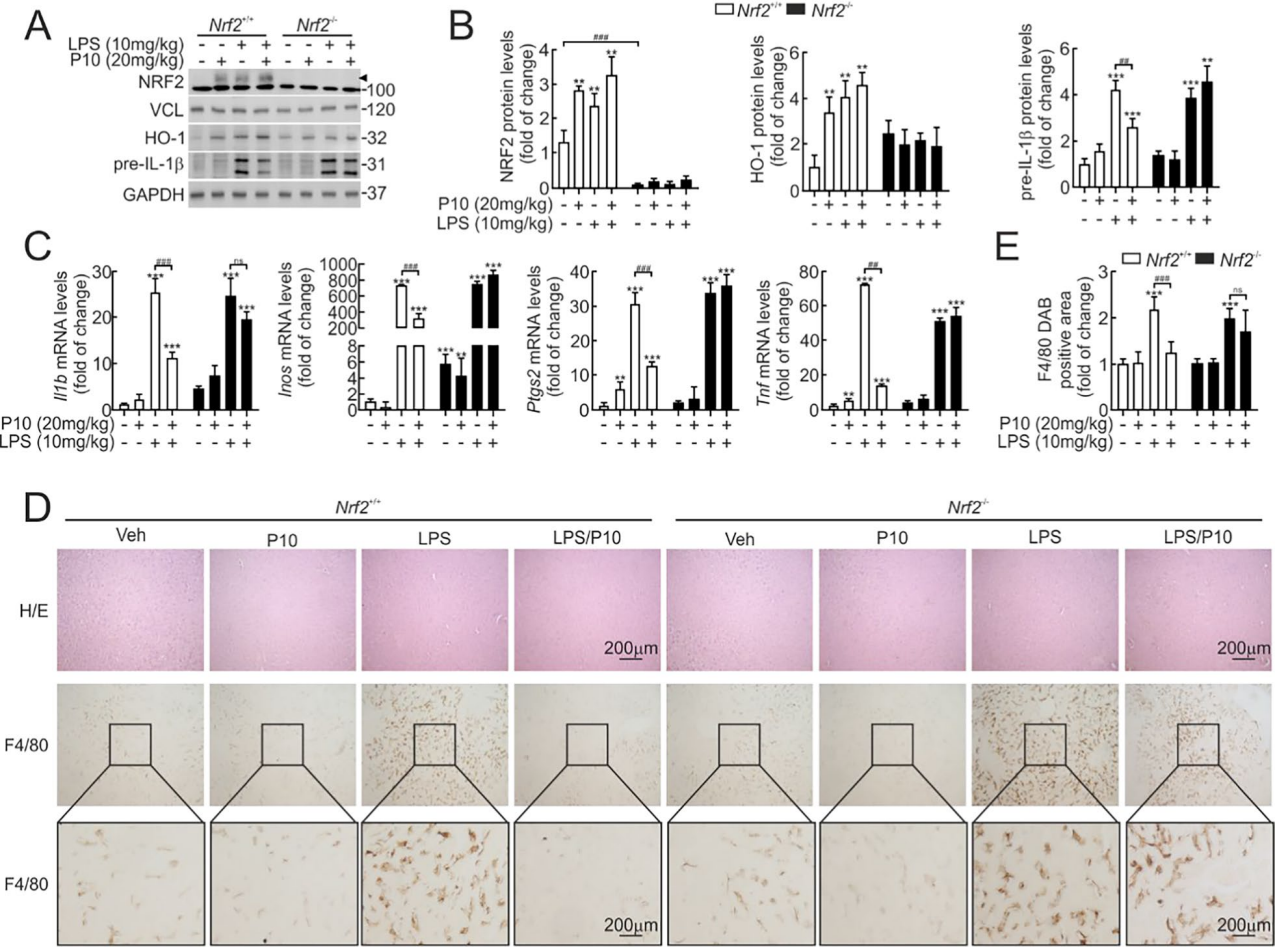
P10 lessens the inflammatory-liver response in the NRF2-dependent context in mice treated with LPS. C57Bl/6 male mice were treated i.p with vehicle (Tween-80 + PBS, 1:7) or 20 mg/kg P10 for 5 days. Two hours after the last administration, mice received vehicle or 10 mg/kg LPS and were sacrificed after 4 h for liver analysis. **A** Representative immunoblots in liver extracts of NRF2 (arrowhead), HO-1, pre-IL-1β, GAPDH, and VCL as a loading control. **B** Densitometric quantification of the NRF2, HO-1, and pre-IL-1β protein levels from representative immunoblots shown in **A**, expressed as a ratio of protein/GAPDH or protein/VCL. Data are mean ± S.D. (n = 5) **p < 0.01, ***p < 0.001 vs. vehicle and ##p < 0.01 vs. LPS according to a Student's t-test. **C** mRNA levels of *Il1b*, *Inos*, *Tnf*, and *Ptgs2* were determined by qRT-PCR and normalized by the geometric mean of *Gapdh* and *Vcl* levels. Data are mean ± S.D. (n = 5). **p < 0.01, ***p < 0.001 vs. vehicle and ##p < 0.01, ###p < 0.001 vs. LPS according to a Student's t-test. **D** Paraffin-embedded liver section stained with H&E and immunohistochemistry for F4/80. **E** Quantification of the DAB-staining positive area of F4/80. Data are mean ± S.D. (n = 5). ***p < 0.001 vs LPS according to a Student's t-test

We assessed whether P10 could prevent LPS-induced inflammation in *Nrf2*⁺/⁺ and *Nrf2*⁻/⁻ mice. Animals were treated daily via intraperitoneal injection with either vehicle or P10 (20 mg/kg) for five consecutive days. Two hours after the final dose, all mice received an intraperitoneal injection of LPS (10 mg/kg) and were sacrificed four hours later for analysis. As anticipated, NRF2 and HO-1 protein levels were increased under these conditions. P10 reduced the LPS-induced expression of pre-IL-1β protein in *Nrf2*^+/+^ mice (Fig. 8A and B) but not in *Nrf2*^−/−^ mice. Moreover, mRNA levels of four pro-inflammatory cytokines, *Il1β*, *Inos*, *Ptgs2*, and *Tnf*, revealed the expected induction by LPS in vehicle-treated mice, which was significantly mitigated in P10-treated *Nrf2*^+/+^ mice, but not in *Nrf2*^−/−^ mice (Fig. 8C).

To gain further information, we further examined H&E-stained liver sections and found that P10 administration did not alter hepatic architecture in either *Nrf2* genotype (Fig. 8D). Immunohistochemical staining with anti-F4/80 of hepatic macrophages (Kupffer cells) revealed that LPS significantly increases staining of this cell type in *Nrf2*^+/+^ mice. As expected, this effect was greatly diminished in P10-treated mice (Fig. 8D and E). Conversely, these inflammatory parameters were not reduced upon P10 treatment in *Nrf2*^−/−^ mice (Fig. 8D and E). Therefore, these results confirm that P10 is protective against liver inflammation in an NRF2-dependent manner.

## Discussion

Several studies have demonstrated that NRF2 activators hold promise as preventive and therapeutic agents for conditions associated with redox dysregulation and inflammatory imbalance [47–49]. Current strategies aimed at activating NRF2 primarily focus on targeting KEAP1 using electrophilic drugs, which form covalent adducts with sulfhydryl groups at specific cysteine residues in KEAP1, thereby inhibiting NRF2 degradation [50, 51]. Another therapeutic approach involves disruption of KEAP1/NRF2 interaction with non-covalent binders [27, 52]. However, drug development pipelines following this strategy are still emerging, and it remains unclear whether these inhibitors may also affect other KEAP1-regulated substrates. Furthermore, the therapeutic window of NRF2 activation via KEAP1/NRF2 inhibition must be carefully considered. For instance, two well-established electrophilic activators of NRF2, DMF and omaveloxolone, can produce some extent of liver injury. DMF increases liver aminotransferase and bilirubin levels in serum just a few days after the first administration [53]. On the other hand, patients over-treated with omaveloxolone experience elevation in hepatic transaminases and B-type natriuretic peptide (BNP), as well as lipid abnormalities [54]. Although KEAP1 inhibitors are well known for their ability to strongly activate NRF2 and trigger a rapid cytoprotective response, excessive or prolonged NRF2 activation can be detrimental. In various cancers, NRF2 is constitutively upregulated, promoting tumor cell survival by enhancing antioxidant defenses and facilitating resistance to therapy. Consequently, sustained or high-level inhibition of KEAP1 may inadvertently contribute to tumor progression, particularly in the setting of established malignancies [55–57]. In other clinical trials, NRF2 hyperactivation has been linked to the development of bone hypoplasia [58], hydronephrosis [59], esophagus, and forestomach hyperkeratosis [60], altered mitochondrial bioenergetics, hallmark features of type 1 diabetes, and accelerated aging [61].

Conversely to the KEAP1/NRF2 interaction, disruption of the β-TrCP1/NRF2 represents an alternative strategy for three main reasons: (1) the interaction between NRF2 and β-TrCP1 is relatively weak compared to other β-TrCP1 substrates [32], which provides a selective window to design PPI-inh that selectively displace NRF2 without broadly affecting other β-TrCP1-regulated proteins; (2) NRF2 activation via β-TrCP1 inhibition is milder than via KEAP1 inhibition [40], and therefore is more likely to remain within physiologically homeostatic levels, reducing risks associated with hyperactivation; and (3) Unlike the KEAP1/NRF2 axis, somatic mutations disrupting the β-TrCP1/NRF2 interaction have not been reported in cancer, suggesting that this alternative pathway might be safer in terms of oncogenic potential. Several chemical compounds reported in the literature can disrupt the interaction between β-TrCP1 and its substrates [62–64]; however, these studies did not analyze the effect on the NRF2 signature. One such compound, GS143, was identified as an inhibitor of IκBα ubiquitination. Nevertheless, its activity does not lead to the accumulation of other β-TrCP1 substrates, including β-CATENIN [63, 65]. A natural product, erioflorin, inhibits the interaction between the tumor-suppressive PDCD4 protein and β-TrCP1 [64]. Previously, we introduced PHAR, a PPI-inh targeting β-TrCP1/NRF2, which induces mild NRF2 activation in the liver and did not seem to modify the levels of other known key substrates of β-TrCP1, such as β-CATENIN, YAP/TAZ, IκBα, and GLI2, suggesting a preference for inhibition of the β-TrCP1/NRF2 interaction [32].

P10 was identified through an ongoing search for alternative disruptors of β-TrCP1/NRF2. Various web servers assist in predicting ADMET properties, such as SwissADME (Swiss Institute of Bioinformatics, Switzerland, http://www.swissadme.ch/) or Molsoft (Molsoft LLC, USA, http://molsoft.com/mprop/) were used to assess the pharmacokinetics and drug-likeness parameters of P10 [66]. Compared to PHAR, P10 presents some advantages. P10 meets all Lipinski’s criteria [67] with zero violations, while PHAR fails in one due to its molecular weight (PHAR: ~ 554.98 Da vs. P10: ~ 455.46 Da). Larger molecules may have difficulty crossing cell membranes and being absorbed in the intestine. This difference may make P10 improve its absorption across biological barriers, such as the gastrointestinal tract and cytoplasmic membranes, than PHAR. Additionally, P10 may offer another slight advantage over PHAR in vivo. A dose of 20 mg/kg of P10 was sufficient to reduce pro-inflammatory markers in the liver of mice subjected to acute inflammation induced by lipopolysaccharide (LPS). In contrast, 50 mg/kg PHAR was required under the same conditions to achieve a similar reduction in LPS-induced pro-inflammatory markers in the liver [32].

P10-mediated activation of NRF2 target gene expression was observed in KEAP1-knockout cells, further confirming a KEAP1-independent mechanism of NRF2 activation. Additionally, P10 activated NRF2 in various cell lines under low-serum growth conditions (16 h, 1% FBS), including MCF-7, MEFs, RAW264.7, and primary peritoneal macrophages. We attribute this effect to the level of GSK-3β activity in each of these cell lines, as this is a crucial requirement for the formation of the NRF2 phosphodegron, which is recognized by β-TrCP1.

Moreover, in the presence of the PI3K inhibitor LY294002, which maintains GSK-3β in its non-phosphorylated active form, leading to NRF2 phosphorylation and subsequent β-TrCP1-mediated degradation, NRF2 levels decreased as expected (Fig. 3D and [68, 69]). However, P10 slightly increased NRF2 levels. Conversely, pharmacological inhibition of GSK-3 by SB216763 increased NRF2 protein levels (Fig. 3F and [68]), and rendered cells unresponsive to P10, supporting the notion that P10 targets the β-TrCP1/NRF2 interaction when NRF2 is phosphorylated by GSK-3β. Taken together, these findings suggest that P10 exhibits a similar mechanism of action to PHAR, as previously described [32].

To further evaluate the mechanistic profile of P10, we investigated whether its ability to induce NRF2 is mediated by electrophilic activity, specifically through modulation of the PI3K/AKT/GSK-3β signaling axis via PTEN regulation. PTEN, a phosphatase that antagonizes PI3K signaling, is known to repress NRF2 activity [70]. Its enzymatic function can be inhibited through redox-sensitive modifications, including oxidation [71], S-sulfhydration [72] of its catalytic cysteine residue Cys124, and S-nitrosylation of the allosteric Cys83 residue [73]. Electrophilic compounds, such as tBHQ, can target these cysteines, forming covalent adducts and thereby inactivating PTEN [39, 74], which leads to downstream NRF2 stabilization.

To test whether P10 behaves similarly, we analyzed its effect on the PI3K/AKT/GSK-3β pathway. Unlike tBHQ, P10 did not alter the phosphorylation status of key pathway components in vivo (Suppl. Fig. S2), indicating that it does not modulate redox-sensitive enzymes. These results support the conclusion that P10 activates NRF2 through a non-electrophilic mechanism. In parallel, we assessed whether P10 influences the MAPK signaling cascade, which can also be affected by electrophilic compounds. Although tBHQ is classified as a phenolic antioxidant, previous studies have shown it can exhibit pro-oxidant activity [75] and activate MAPK pathways, including ERK1/2, JNK, and p38 [76]. Our data confirmed that tBHQ robustly induces MAPK activation (Suppl. Fig. S2). In contrast, P10 did not affect MAPK signaling, further confirming the absence of pro-oxidant behavior. Together, these findings reinforce that P10 functions as a non-electrophilic NRF2 activator, mechanistically distinct from classical agents like tBHQ.

Notably, while P10 prevents NRF2 degradation and increases its protein levels, this effect is not observed in another β-TrCP1 substrate, β-CATENIN (Fig. 4A). This effect has also been reported for GS143. This molecule disrupts the interaction between β-TrCP1 and IκBα but does not affect β-CATENIN [65]. One possible explanation may stem from a different mode of docking of different substrates to β-TrCP, resulting in different affinities. MNR studies indicated that phosphorylated NRF2 binds to β-TrCP at key amino acid residues, including Arg^474^, Arg^524^, Leu^351^, Asn^394^, and Ala^434^ [29]. In contrast, the crystal structure of β-CATENIN binding to β-TrCP1 suggests that this protein binds β-TrCP in a different manner using a different set of amino acids, such as Arg^285^, Tyr^271^, Arg^431^, Arg^474^, and Tyr^488^ [45]. Our in silico analysis suggests that P10 only interacts with three of these residues (Tyr^271^, Arg^474^, and Tyr^488^), and it does not seem to fully disrupt the β-TrCP1/β-CATENIN interaction. Contrary to P10 and GS143, erioflorin is a less selective inhibitor of β-TrCP1, since it interferes with the interaction between β-TrCP1 and PDCD4 but also other β-TrCP1 substrates, stabilizing and preventing ubiquitination of the IκBα and β-CATENIN [64]. Thus, P10 functions as a selective PPI-inh of β-TrCP1 that may be more specific for NRF2 than for other substrates.

Although we did not directly assess the effect of P10 on other classical β-TrCP1 substrates such as IκBα, our findings provide indirect evidence of its specificity. In models of LPS-induced inflammation—including peritoneal macrophages and mice—P10 failed to modulate pro-inflammatory markers in the absence of NRF2. This indicates that its anti-inflammatory effects are largely dependent on NRF2 activation rather than off-target inhibition of other β-TrCP1 substrates like IκBα. These observations support the selective action of P10 in disrupting the β-TrCP1/NRF2 interaction without broadly affecting other β-TrCP1-regulated pathways.

In vivo β-TrCP1/NRF2 ubiquitination assay provided evidence that P10 acts as an inhibitor of NRF2 ubiquitination, supporting its role as a PPI-inh. Additionally, the co-immunoprecipitation assay demonstrated that P10 disrupts the physical interaction between β-TrCP1 and NRF2, exhibiting a behavior similar to that of the β-TrCP1-binding mutant of NRF2.

Upon intraperitoneal (i.p.) administration of P10, its biological activity was detected in the liver, which is our primary interest for future studies in liver diseases. However, other routes of administration might be explored to target other organs. For instance, GS143, administered intranasally before the antigen challenge, effectively suppressed antigen-induced NF-κB activation in the lungs of sensitized mice [63]. This suggests that intranasal administration could be a potential route for P10 treatment of lung or neurological diseases in future studies.

Thus, we demonstrate that P10 mediates an NRF2-dependent downregulation of inflammation in both in vitro and in vivo models. This effect is likely due to its ability to disrupt the interaction between β-TrCP1 and inflammatory signaling pathways linked to NRF2 activation. Targeting the liver with P10 could be a promising strategy for treating various inflammatory liver diseases.

## Conclusions

This study identifies P10 as a novel small-molecule inhibitor that selectively disrupts the protein–protein interaction between NRF2 and β-TrCP1, offering a mechanistically distinct alternative to conventional KEAP1-targeting NRF2 activators. Unlike many electrophilic KEAP1 inhibitors, which can lead to excessive or prolonged NRF2 activation and associated adverse effects, P10 activates NRF2 through a non-electrophilic and β-TrCP1-dependent mechanism, resulting in a more moderate and potentially safer therapeutic profile. Our findings demonstrate that P10 effectively stabilizes NRF2, enhances the expression of its cytoprotective targets, and confers anti-inflammatory benefits in both cellular and murine models of acute liver inflammation. Importantly, these effects are absent in *Nrf2*-null systems, reinforcing the compound’s NRF2 specificity. Future research will focus on detailed toxicity assessments, pharmacokinetic and pharmacodynamic profiling, and preclinical efficacy studies across relevant disease models. Collectively, our results position P10 as a promising next-generation NRF2 activator with a unique mode of action and strong potential for clinical development in the treatment of inflammatory liver disorders.

## Supplementary Information

Additional file 1.

Additional file 2.

## Data Availability

The datasets used and/or analyzed during the current study are available from the corresponding author upon reasonable request.

## Abbreviations

ADMET: Absorption, distribution, metabolism, excretion and toxicity
AKT: Protein kinase B (PKB)
ARE: Antioxidant response element
β-TrCP: β-transducin repeat-containing protein
COX-2: Cyclooxygenase-2
CD36: Cluster of differentiation 36
CUL3: Cullin-3
DHE: Dihydroethidine
DMF: Dimethyl fumarate
ERK: Extracellular signal-regulated kinase
FBS: Fetal bovine serum
GAPDH: Glyceraldehyde-3-phosphate dehydrogenase
GSK-3β: Glycogen synthase kinase-3β
HO-1: Heme oxygenase 1
HPLC: High-performance liquid chromatography
IL-1β: Interleukin-1 beta
IL-17D: Interleukin-17D
IL-6: Interleukin-6
iNOS: Inducible NOS
JNK: C-Jun N-terminal kinases
KEAP1: Kelch-like ECH-associated protein 1
LPS: Lipopolysaccharide
MAF: Musculoaponeurotic fibrosarcoma oncogene homolog
MAPKs: Mitogen-activated protein kinases
MCF-7: Michigan cancer foundation-7
MEF: Mouse embryonic fibroblasts
MMP-9: Matrix metalloproteinase-9
NF-κB: Nuclear Factor kappa-light-chain-enhancer of activated B cells
NQO1: NAD(P)H: quinone oxidoreductase 1
NRF2: Nuclear factor erythroid 2‐Related Factor 2
PI3K: Phosphoinositide 3-Kinase
PPI-inh: Protein–protein interaction-inhibitor
PTEN: Phosphatase and Tensin homolog deleted on chromosome 10
RBX1: RING-box protein 1
SAPK: Stress-activated protein kinases
SFN: Sulforaphane
tBHP: Tert Butyl hydroperoxide
TNFα: Tumor necrosis factor-alpha
VCAM-1: Vascular cell adhesion molecule-1
VCL: Vinculin
