# Supplementary material for: A novel β-TrCP1/NRF2 interaction inhibitor for effective anti-inflammatory therapy
Source: J Biomed Sci. 2025 Jul 11;32:65. doi: 10.1186/s12929-025-01157-3 (PMC12247323; doi:10.1186/s12929-025-01157-3)

**A Novel β-TrCP1/NRF2 Interaction Inhibitor for Effective Anti-Inflammatory Therapy**

Ángel J. García-Yagüe^1,2,3,4 *^, Lucía Cañizares-Moscato^1,2,5^, José Antonio Encinar^6^, Eduardo Cazalla^1,2,3,4^, Raquel Fernández-Ginés^1,2,3,4^, Maribel Escoll^1,2,3,4^, Ana I. Rojo^1,2,3,4^ and Antonio Cuadrado^1,2,3,4*^

**supplementary material**

**Suppl. figures**

Figure S1


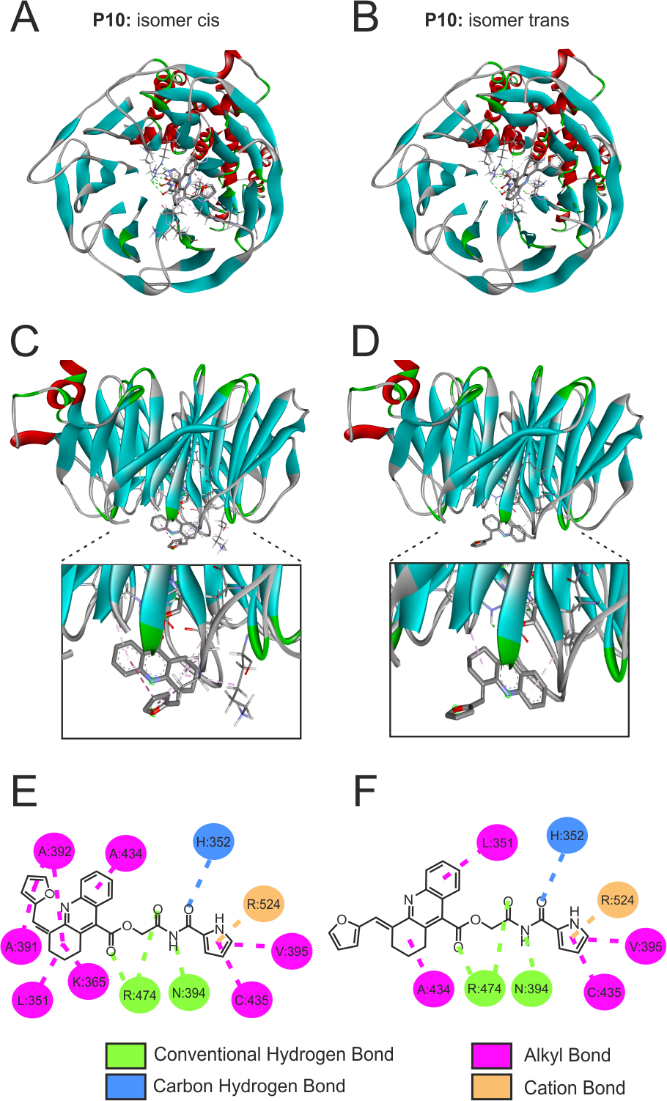


**Figure S1. Representative models of the interaction between P10 and the WD40 propellers of the β-TrCP1. (**A-B) P10 docked on the interaction tertiary-structure of β-TrCP1 with both isomers, respectively of β-TrCP1 (PDB ID:1P22). (C-D) Sagittal projection of the WD40 domain of β-TrCP1 binds to both isomers of P10. Magnification of the interaction of P10 with the β-propeller of the WD40 domain. (E-F) The spatial location of the amino acids of β-TrCP1 that maintain hydrophobic or electrostatic interactions with both isomers of the P10 compound.

Figure S2

**
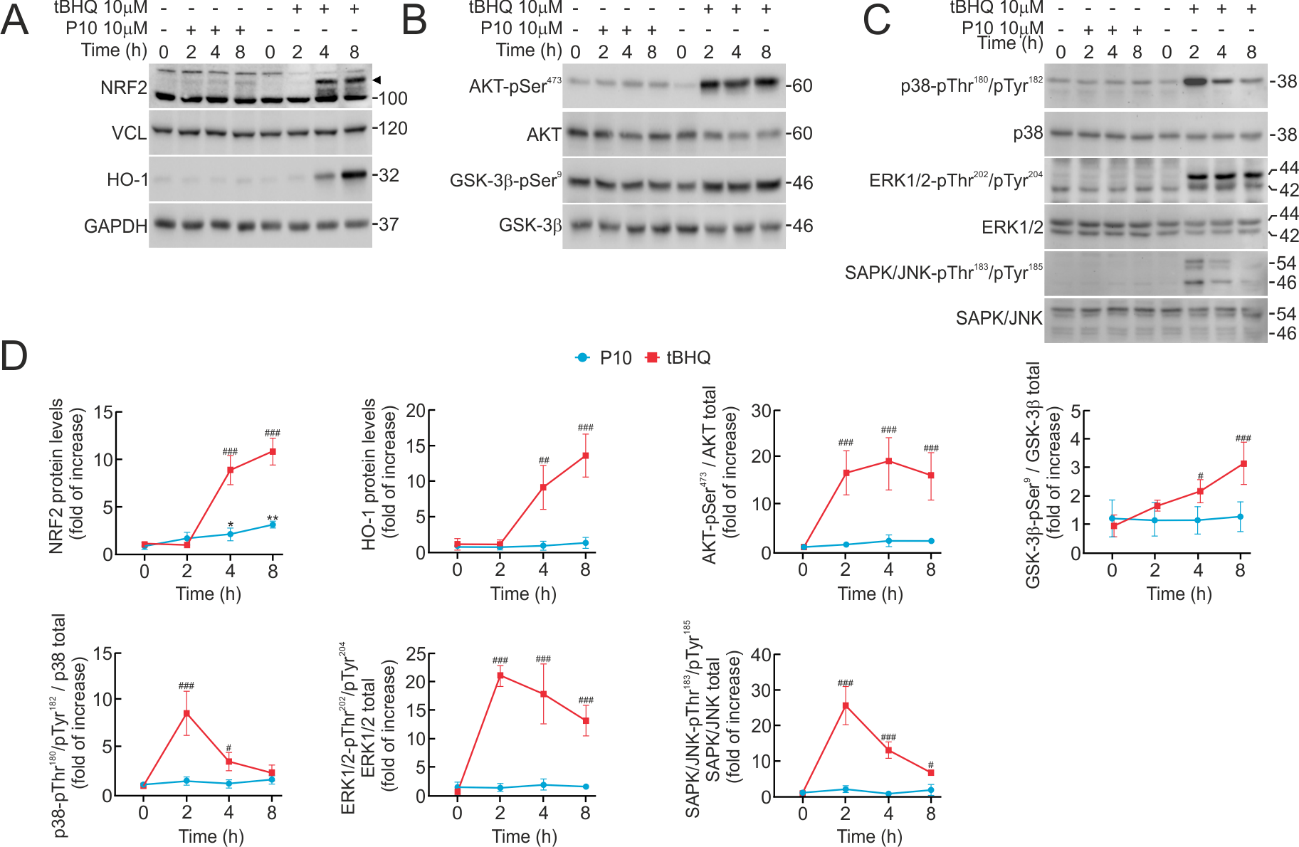
**

**Figure S2. P10 doesn’t regulate the signaling through PI3K/AKT and MAPK pathways.** (A) Representative immunoblots of NRF2 (arrowhead), HO-1, GAPDH, and VCL as a loading control from low-serum starved (16 h, 1% FBS). (B) Representative immunoblots of AKT-pSer^473^, AKT, GSK-3β-pSer^9,^ and GSK-3β. (C) Representative immunoblots of MAPK pathways p38-pThr^180^/pTyr^182^, p38, ERK1/2-pThr^202^/pTyr^204^, ERK1/2, SAPK/JNK-pThr^183^/pTyr^185^, and SAPK/JNK. MCF-7 c32^ARE-LUC^ were submitted to 10 μM P10 or 10 μM tBHQ for the indicated times. (D) Densitometric quantification of NRF2, and HO-1 protein levels from representative immunoblots of A normalized with GAPDH and VCL, AKT-pSer473, GSK-3β-pSer9 protein levels from representative immunoblots of B normalized with total AKT and GSK-3β, and p38-pThr^180^/pTyr^182^, ERK1/2-pThr^202^/pTyr^204^ and SAPK/JNK-pThr^183^/pTyr^185^ protein levels from representative immunoblots of C normalized with total p38, ERK1/2 and SAPK/JNK, respectively. Data are mean ± S.D. (n = 3). *p < 0.05; **p < 0.01, ##p < 0.01, ###p < 0.001 vs. point 0 according to a one-way ANOVA test.

Figure S3

**
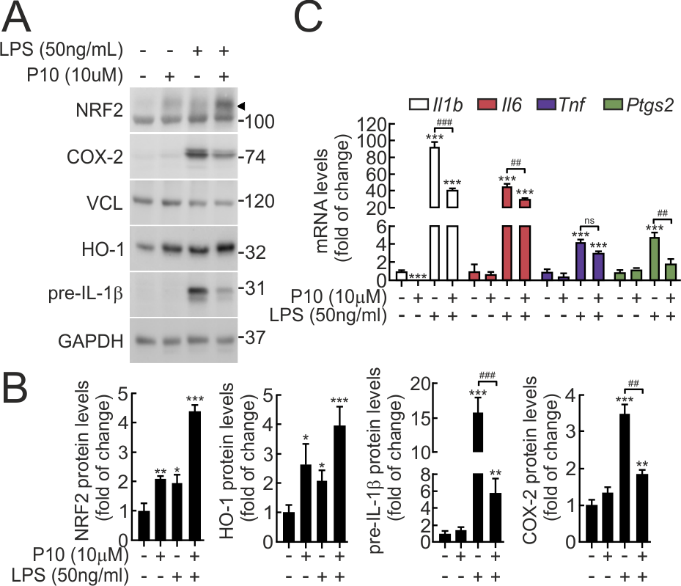
**

**Figure S3. P10 decreases the inflammatory response in Raw264.7 cells stimulated with LPS.** (A) Low-serum starved (16 h, 1% FBS) Raw264.7 cells were pre-treated with 10 μM of P10 for 16 h. Then, cells were treated with 100 ng/ml of LPS for 3 h. (A) Representative immunoblots of NRF2 (arrowhead), HO-1, pre-IL-1β, COX-2, GAPDH, and VCL as a loading control. (B) Densitometric analysis of NRF2, pre-IL-1b, COX-2, and HO-1 protein levels from a representative immunoblot from A, normalized with GAPDH and VCL. Data are mean ± S.D. (n = 3). *p < 0.05; **p < 0,01; ***p < 0,001 vs vehicle or ##p < 0.01, ###p < 0.001 vs. LPS according to a Student's t-test. (C) mRNA levels of *Il1b*, *Ptgs2*, *Il6*, and *Tnf* were determined by qRT-PCR and normalized by *Gapdh* and *Vcl* average. Data are mean ± S.D. (n = 3). ***p < 0.001 vs vehicle or ##p < 0.01, ###p < 0.001 vs. LPS according to a Student's t-test.

**Suppl. Materials and Methods**

**Structure modeling of receptor-ligand interaction.** Representation of β-TrCP1 (PDB ID: 1P22) interaction with the P10 compound ribbon-cartoon and its prediction of interactions with amino acidic residues from the substrate recognition site (Suppl. Fig. S1), was performed with an additional molecular docking using the script Autodock Vina from VEGA ZZ 3.2.1 (77). The three-dimensional structure of the ligand was prepared from the Simplified Molecular-Input Line-Entry System (SMILES) and minimized to the optimal conformation through steepest descent, conjugated gradients, and truncated Newton algorithms using VEGA ZZ. Receptor proteins were prepared from the PDB files in Discovery Studio by delimiting the binding site to obtain grid dimensions, coordinates, and removing any native ligand groups and water molecules. Discovery Studio Visualizer 2020 (DSV, v20.1.0.19295) was used to visualize the docking poses and identify the receptor-ligand interactions.

**Ubiquitination assay.** After five hours of transfection, MCF-7 c32^ARE-Luc^ cells were treated with 1μM MG132 (Sigma) to block protein degradation along with vehicle or P10 to different concentrations: 1 μM, 3 μM, and 9 μM for 16 h. Cells were, then, lysed in a RIPA buffer (150 mM NaCl, 25 mM Tris-HCl, pH 7.5, 1% Nonidet P-40, 1% sodium deoxycholate, 1% Triton-X100, 0.1% SDS, 1mM phenylmethylsulfonyl fluoride, 1mMNaF, 1 mM sodium pyrophosphate, 1 mM sodium orthovanadate, 1 g/ml leupeptin). After that, the samples were kept for 30 min at 4°C in a rotating wheel and centrifuged at 13000 rpm for 10 min. The lysate was incubated with 20μl of Probond TM resin (Invitrogen, Carsband, CA), and the mixture was rotated for 1h at 4°C. A lysate from HA-Ub non-transfected cells was used to control for non-specific ubiquitin, and another lysate from non-transfected cells with pcDNA3-Flag-β-TrCP1 plasmid to control for non-specific binding. The complexes were harvested by centrifugation and washed three times with RIPA buffer. Pull-down complexes were eluted in sample buffer by boiling, electrophoresed through SDS–PAGE gels, and subjected to immunoblot analysis.

**Co-immunoprecipitation assay.** After 5 h, MCF-7 c32^ARE-Luc^ cells were treated for 16 h with 1μM MG132 (Sigma) to block protein degradation and either vehicle or P10 10µM. Cells were then lysed in a RIPA buffer (150 mM NaCl, 25 mM Tris–HCl, pH 7.5, 1% Nonidet P-40, 1% sodium deoxycholate, 1% Triton-X100, 0.1% SDS, 1 mM phenylmethylsulfonyl fluoride, 1 mM NaF, 1 mM sodium pyrophosphate, 1 mM sodium orthovanadate, 1 g/ml leupeptin). Then, samples were kept for 30 min at 4 °C in a rotating wheel and centrifuged at 13,000 rpm for 10 min. Two microliters of the anti-FLAG (Sigma-Aldrich) were added per lysate, and after incubation for 2 h at 4 °C in a rotating wheel, gamma-bind Sepharose-protein G was added (Amersham Biosciences), followed by incubation for 1 h at 4 °C. The immunocomplexes were harvested by centrifugation and washed in three washes with RIPA buffer, resolved in SDS–PAGE gels, and immunoblotted. Mouse IgG TrueBlot (eBiosciences) was used as a peroxidase-conjugated secondary antibody (1:10,000 dilution) to minimize interference by the 55-kDa heavy and 23-kDa light chains of the immunoprecipitation antibody.

**SUPPL. Original Western Blots**

**
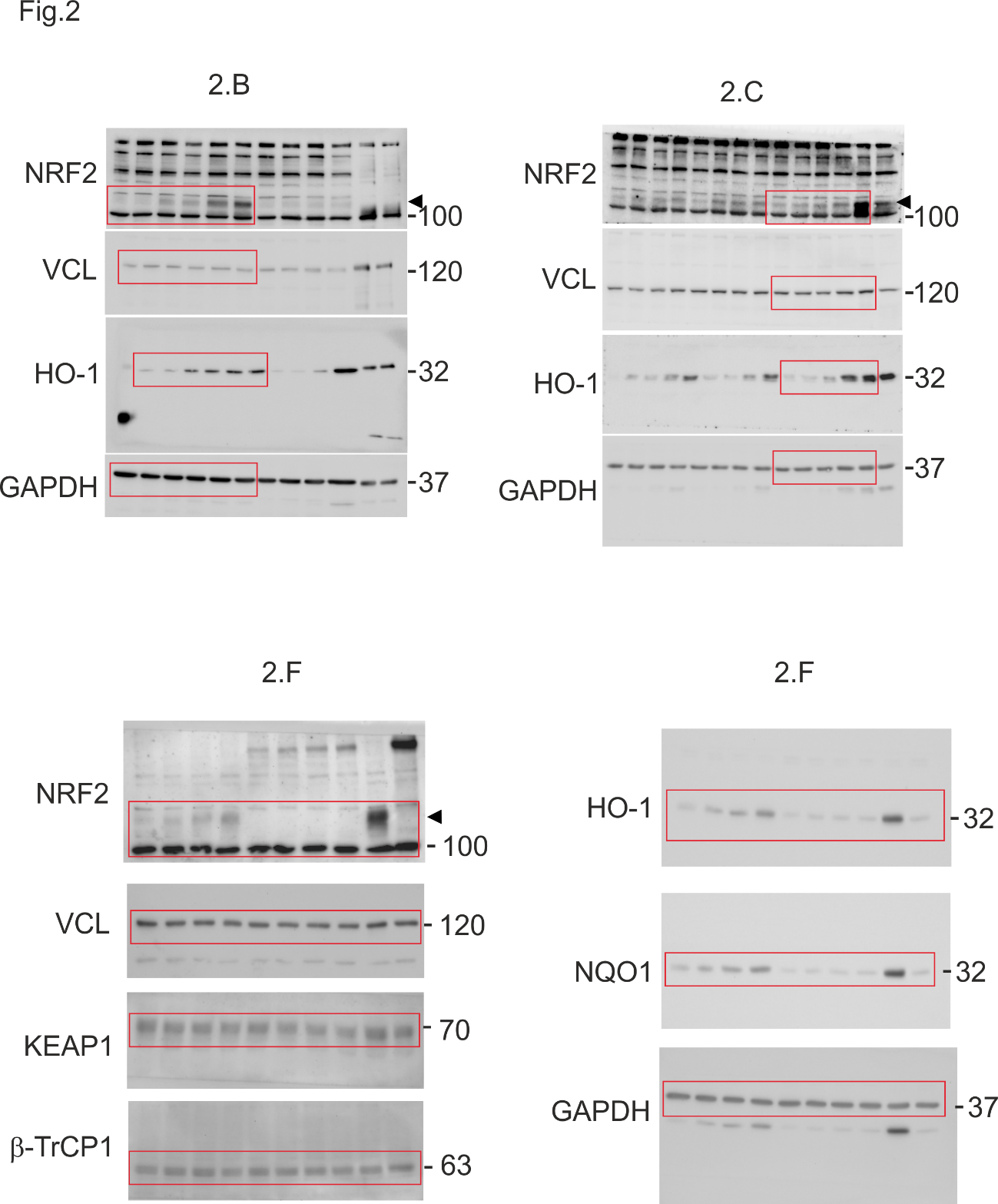
**


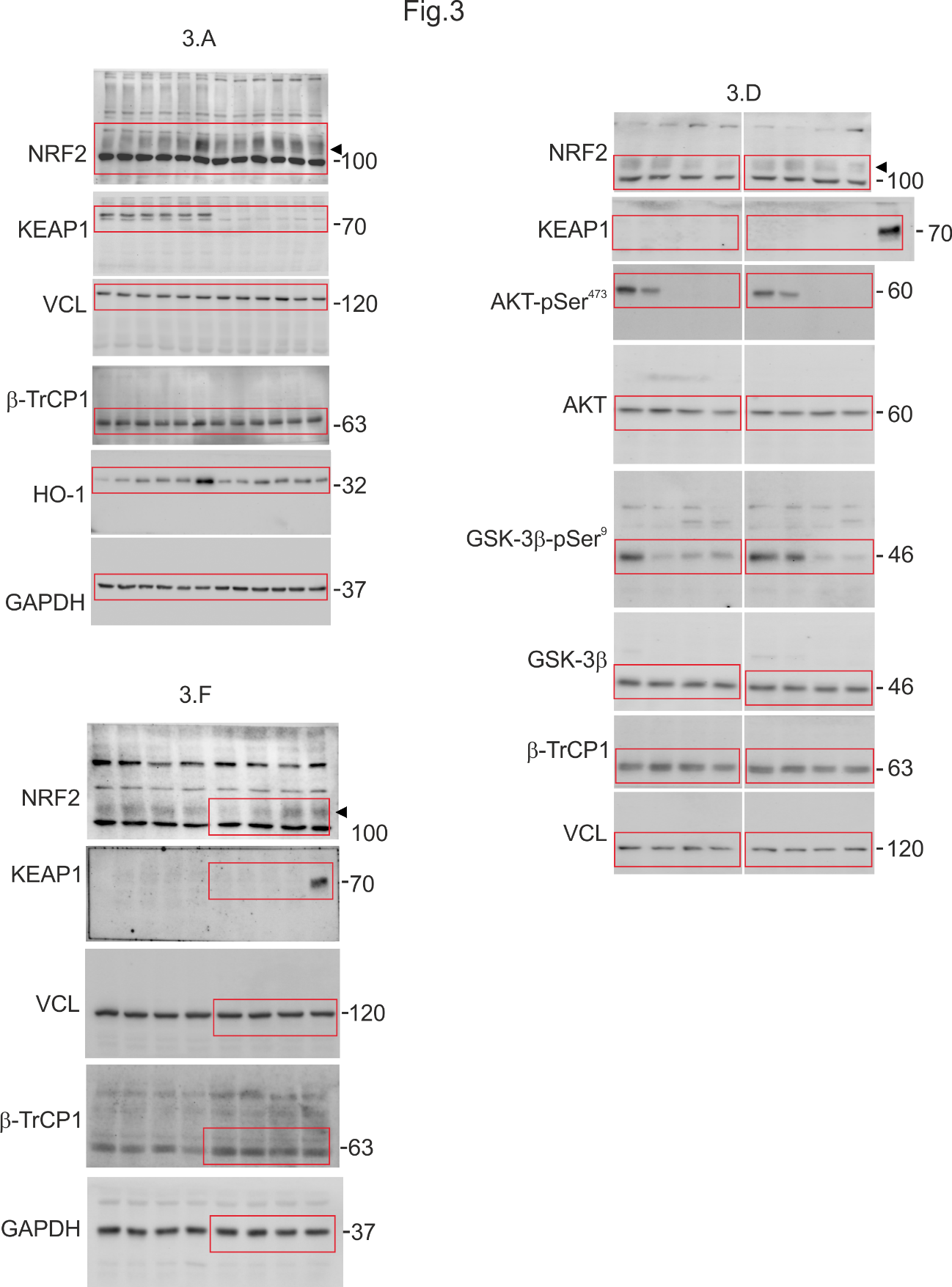


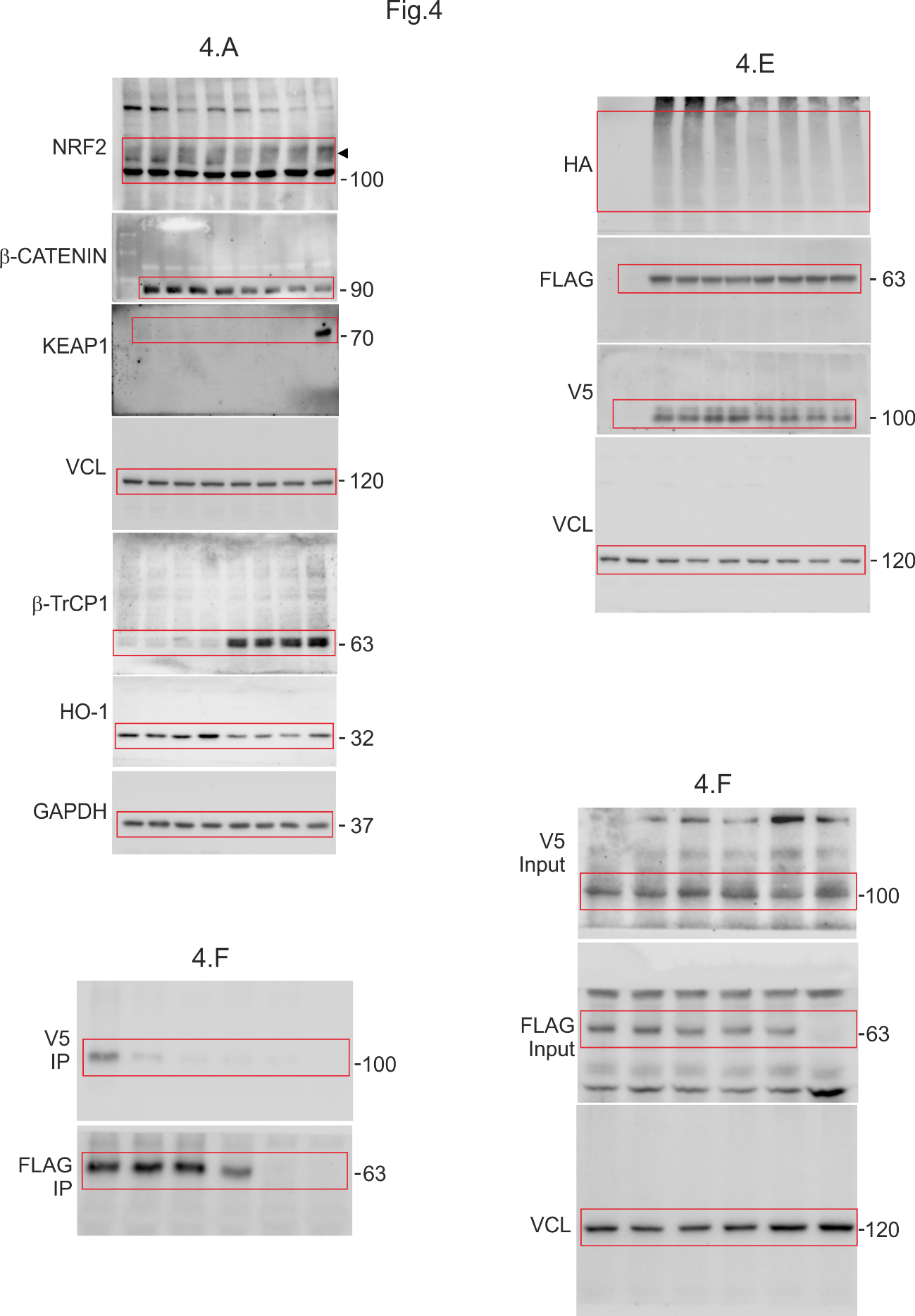


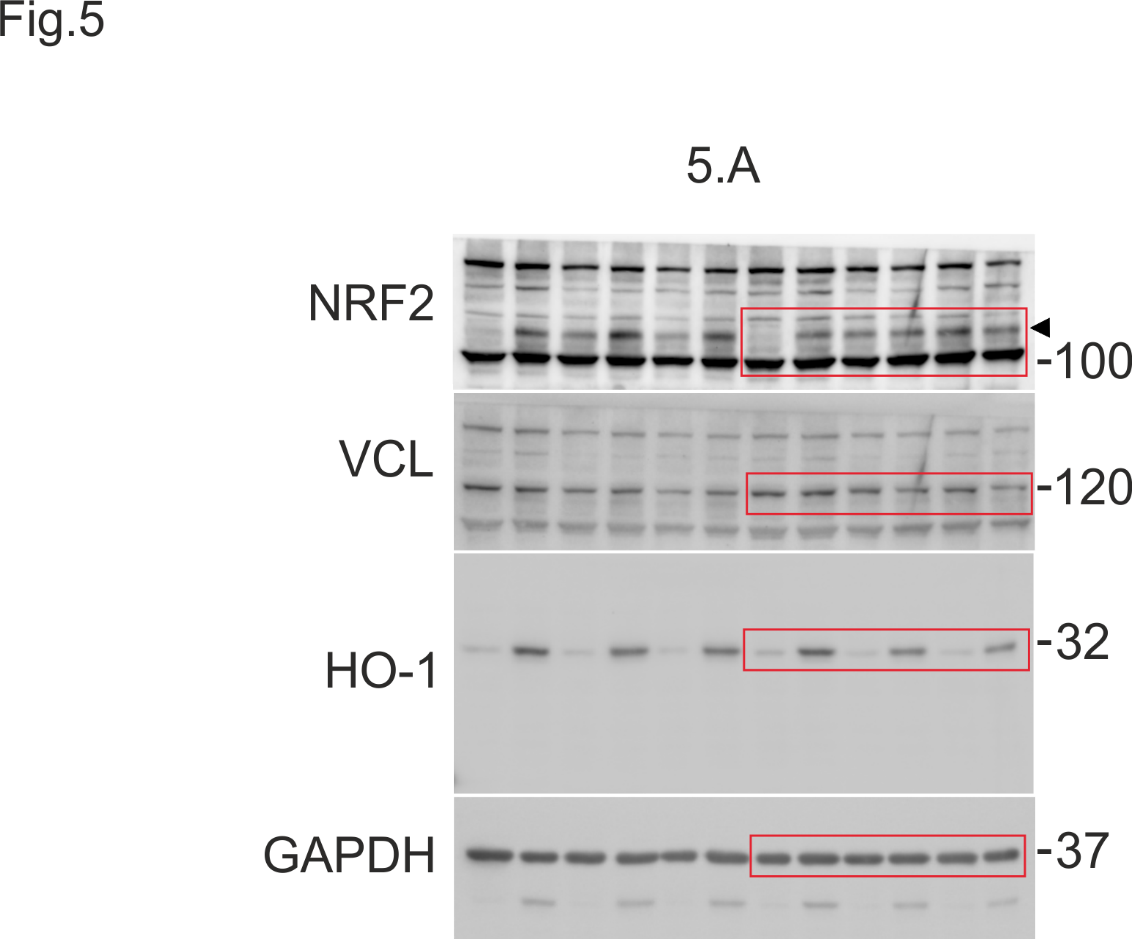


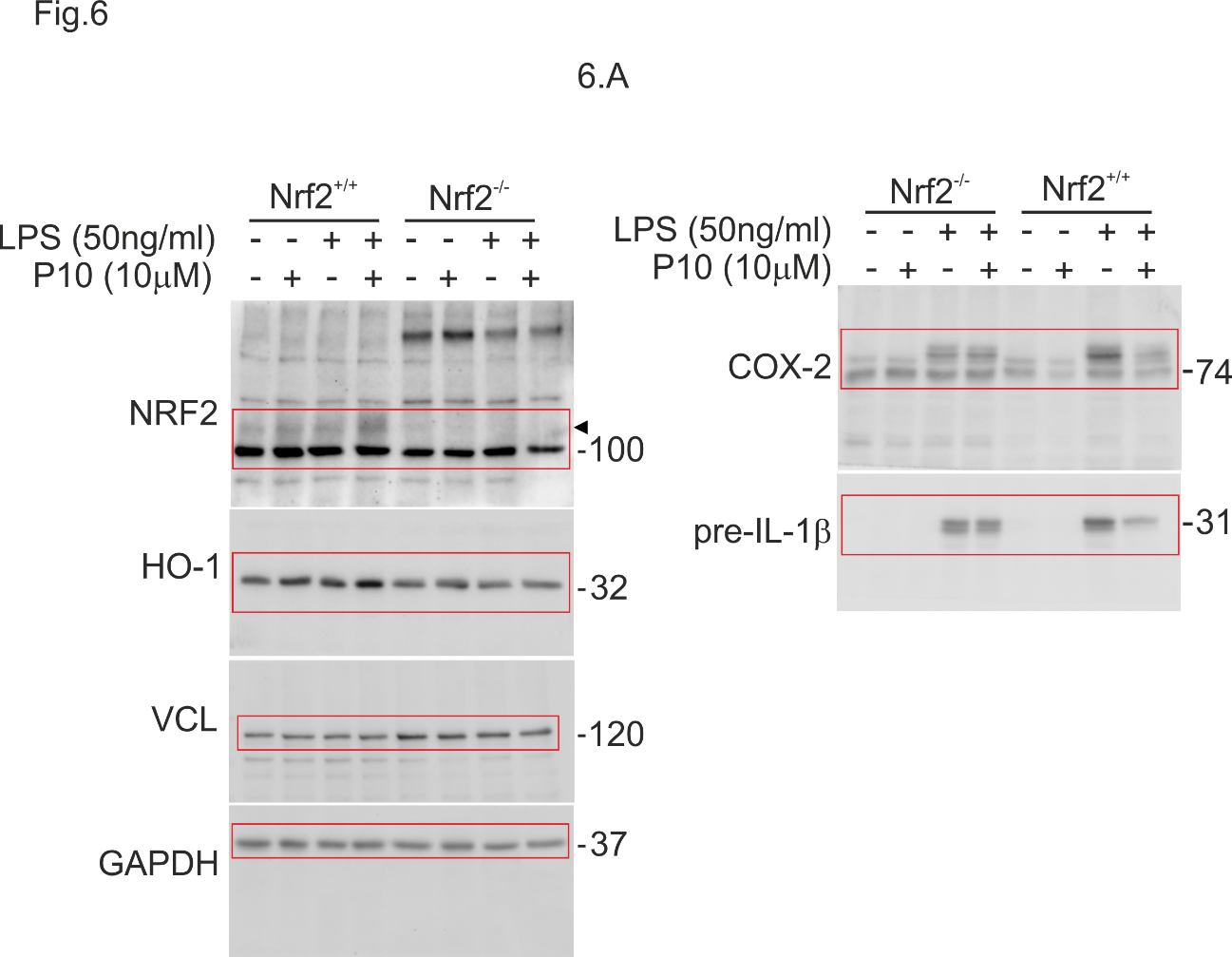


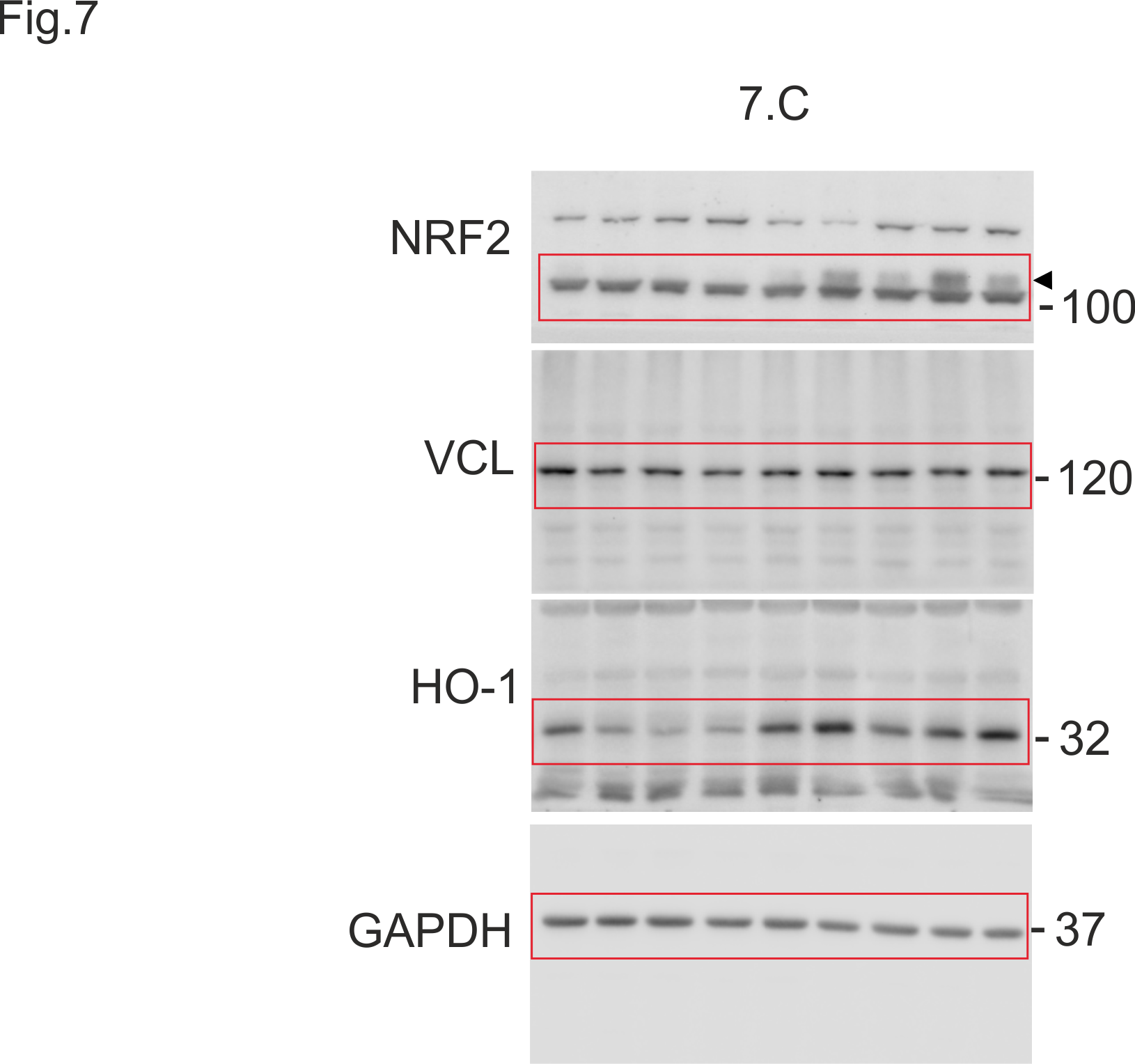


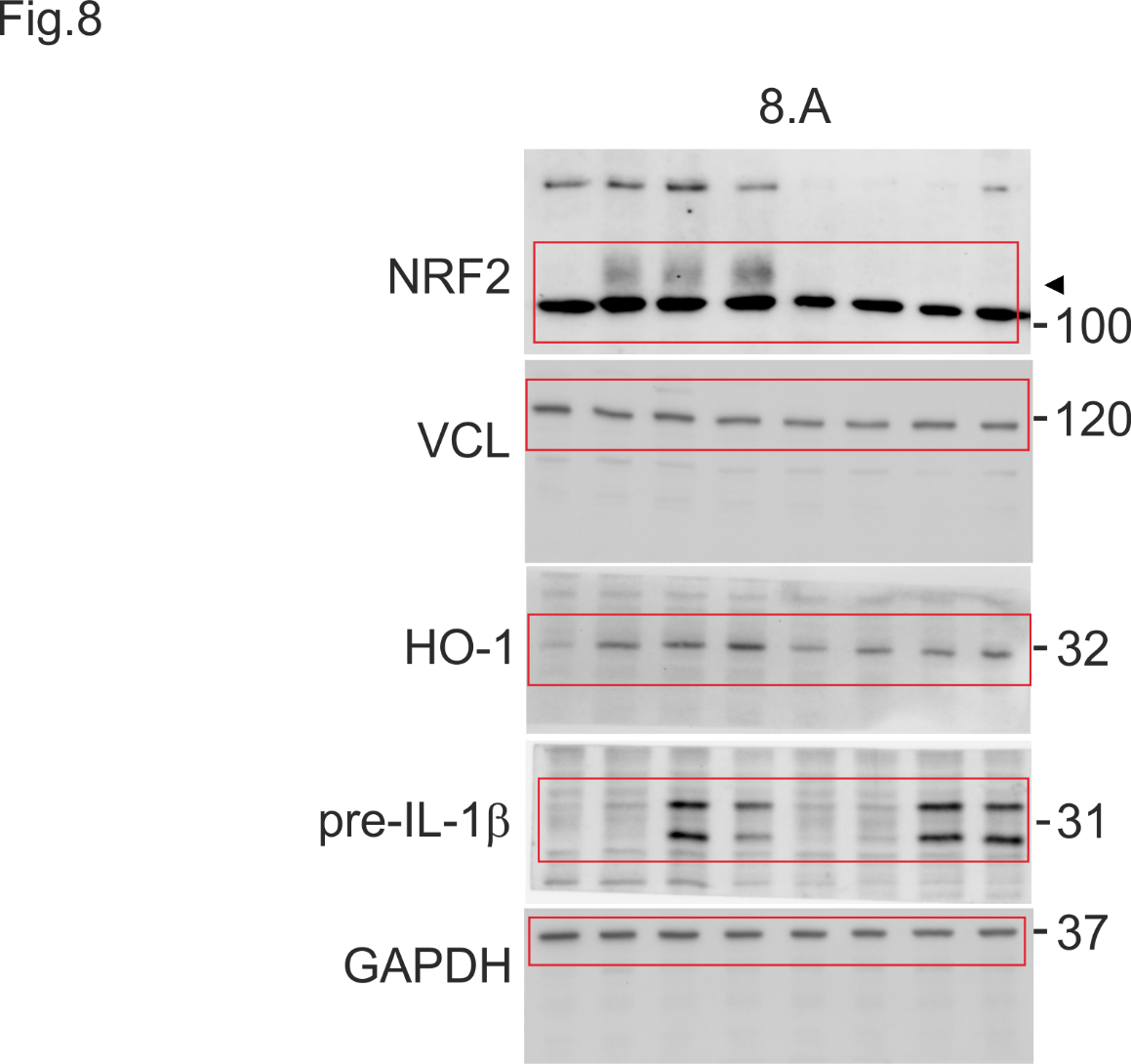


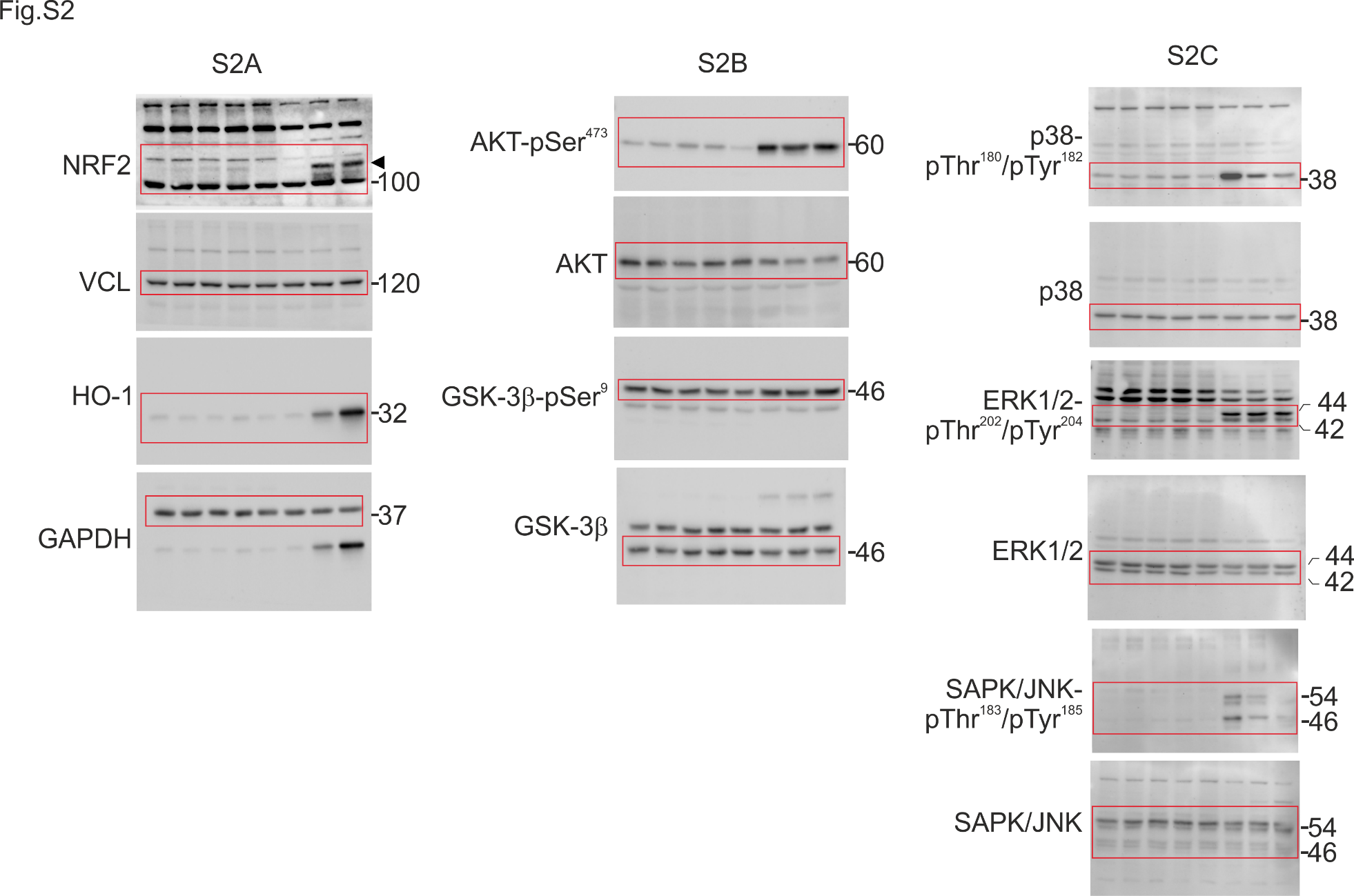


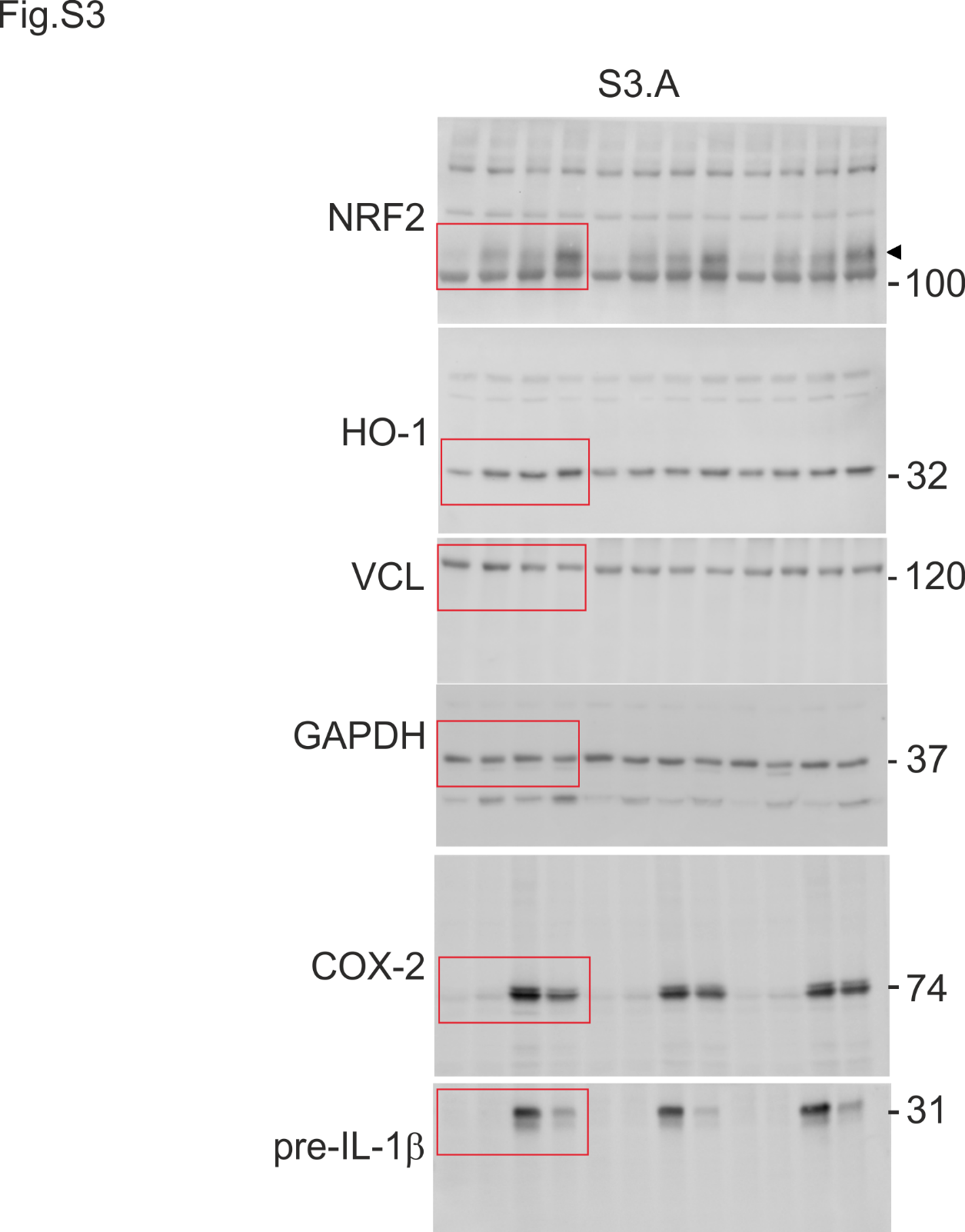

Supplement: Supplementary file 1 — Additional file 1. [file 12929_2025_1157_MOESM1_ESM.docx]
