# Supplementary material for: A novel β-TrCP1/NRF2 interaction inhibitor for effective anti-inflammatory therapy
Source: J Biomed Sci. 2025 Jul 11;32:65. doi: 10.1186/s12929-025-01157-3 (PMC12247323; doi:10.1186/s12929-025-01157-3)
Supplement: Supplementary file 2 — Additional file 2. [file 12929_2025_1157_MOESM2_ESM.docx]

**A Novel β-TrCP1/NRF2 Interaction Inhibitor for Effective Anti-Inflammatory Therapy**

Ángel J. García-Yagüe^1,2,3,4 *^, Lucía Cañizares-Moscato^1,2,5^, José Antonio Encinar^6^, Eduardo Cazalla^1,2,3,4^, Raquel Fernández-Ginés^1,2,3,4^, Maribel Escoll^1,2,3,4^, Ana I. Rojo^1,2,3,4^ and Antonio Cuadrado^1,2,3,4*^

**supplementary material**

**SUPPL. TABLES**

Suppl. Table 1. List of antibodies used in this study.

| **Antibody** | **Source** | **Catalog. Number** |
| --- | --- | --- |
| V5 | Invitrogen | R960-25 |
| FLAG | Sigma-Aldrich | F3165 |
| HA | Covance | MMS-101R |
| VINCULIN | Cell Signaling Technology | E1E9V |
| GAPDH | Merck-Millipore | CB1001 |
| NRF2 | Homemade | Ref. (28) |
| HO-1 | Homemade | Ref. (39) |
| NQO1 | Abcam | ab2346 |
| KEAP1 | Cell Signaling Technology | 80475 |
| β-TrCP1 | Cell Signaling Technology | D13F10 |
| β-CATENIN | Becton Dickinson | 610153 |
| pSer^473^-AKT | Cell Signaling Technology | 4058 |
| AKT | Becton Dickinson | 610860 |
| pSer^9^-GSK-3β | Cell Signaling Technology | 9336 |
| GSK-3β | Becton Dickinson | 610201 |
| COX-2 | Santa Cruz Biotechnology | sc-1747 |
| pre-IL-1β | RD Systems | AF-401-NA |
| p38-pThr180/pTyr182 | Cell Signaling Technology | 9211 |
| p38 | Cell Signaling Technology | 9212 |
| ERK1/2-pThr202/pTyr204 | Cell Signaling Technology | 9106 |
| ERK1/2 | Cell Signaling Technology | 4695 |
| SAPK/JNK-pThr183/pTyr185 | Cell Signaling Technology | 4668 |
| SAPK/JNK | Cell Signaling Technology | 9252 |

Suppl. Table 2. List of primers used for qRT-PCR in this study.

| **Gene** | **5´ Forward primer 3´** | **5´ Reverse primer 3´** | **Species** |
| --- | --- | --- | --- |
| *HMOX1* | TGCTCAACATCCAGCTCTTTGA | GCAGAATCTTGCACTTTGTTGC | Human |
| *SLC7A11* | TGCTGGGCTGATTTATCTTCG | GAAAGGGCAACCATGAAGAGG |  |
| *OSGIN1* | ATGCAGAAGAAGCGAAGAGGT | CCCAGACCCTTCTTGACCAC |  |
| *VCL* | CCCGGCACTCCATGCTTA | ACAAAAACCAGGATATAGGAGACA |  |
| *GAPDH* | CTCTCTGCTCCTCCTGTTCGAC | TGAGCGATGTGGCTCGGCT |  |
|  |  |  |  |
| *Hmox1* | CACAGATGGCGTCACTTCGTC | GTGAGGACCCACTGGAGGAG | Mouse |
| *Nqo1* | GGTAGCGGCTCCATGTACTC | CATCCTTCCAGGATCTGCAT |  |
| *Slc7a11* | TTCATCCGGCACTATTTTC | CGTCTGAACCACTTGGGTTT |  |
| *Osgin1* | CGGTGACATCGCCCACTAC | GCTCGGACTTAGCCCACTC |  |
| *Btrc* | AGCGGCTCCTCTGACAACACCAT | AGCACGGGGTCCAAAGCA |  |
| *Fbxw11* | GTCCGCACTCTGAATGGGCACA | GCACCGGACCAATTCTTCG |  |
| *Cox2* | TTCGGGAGCACAACAGAGTG | TAACCGCTCAGGTGTTGCAC |  |
| *Inos* | CCTCCTTTGCCTCTCACTCTTC | AGTATTAGAGCGGTGGCATGGT |  |
| *Il1b* | CTGGTGTGTGACGTTCCCATTA | CCGACAGCACGAGGCTTT |  |
| *Tnfa* | CATCTTCTCAAAATTCGAGTGACAA | TGGGAGTAGACAAGGTACAACCC |  |
| *Il6* | CCTACCCCAATTTCCAATGCT | TATTTTCTGACCACAGTGAGGAATG |  |
| *Gapdh* | CGACTTCAACAGCAACTCCCACTCTTCC | TGGGTGGTCCAGGGTTTCTTACTCCTT |  |
| *Vcl* | TGTGAAAGCTGCCTCTGATGA | GAAGCTCTTTTGCAGGTCGGG |  |
